# Dual-Channel Sensing and Event-Based Feedback for Cardiopulmonary Resuscitation Training

**DOI:** 10.3390/s26216880

**Published:** 2026-10-30

**Authors:** Haoxiang Zhang, Xinyu Zhu, Heyan Yang, Lijun Yuan, Zishun Zhao

**Affiliations:** College of Avionics and Electrical Engineering, Civil Aviation Flight University of China, Guanghan 618307, China

**Keywords:** cardiopulmonary resuscitation, manikin-based training, strain sensing, airflow switch, event fusion, finite-state machine, embedded feedback

## Abstract

**Highlights:**

**What are the main findings?**

- The prototype characterisation quantifies calibration agreement, compression qualification, airflow detection and electrical feedback timing as distinct stages of CPR feedback.
- Loading, installation and input conditions define the observed operating envelope.

**What are the implications of the main findings?**

- Separating measurements, detected events and feedback decisions identifies which stage requires calibration or further validation.
- The results provide a basis for evaluating fixed calibration parameters, event-matched classification and training outcomes.

**Abstract:**

Coordinated compression and ventilation feedback in manikin-based cardiopulmonary resuscitation (CPR) training requires clear links between sensor measurements, detected events and quality decisions. We characterise these links in an STM32 prototype combining a BF350 strain gauge, an AD620 interface and an HU108 airflow switch. Five static laser-reference pairs used for recalibration yielded a mean system-minus-reference bias of +1.14 mm, MAE of 1.34 mm and RMSE of 1.98 mm. Loading–unloading separation was 0.7–2.3 mm, and refitted conversion coefficients varied with installation. Laser-labelled qualified-compression trials yielded count-based sensitivities of 88.0–93.5% across regular rhythms; false-positive proportions were 46.5% for shallow actions and 20.5% for incomplete release. Controlled forward-flow tests detected 30/30 routine pulses and 3/30 weakest pulses, with 99.89% specificity in the defined one-second no-flow windows. Across 100 electrical input-to-LED-driver tests, mean delay was 26.4 ms and the observed range was 17.5 ms. These results define an operating envelope for the prototype and identify calibration, action qualification and airflow intensity as distinct determinants of feedback reliability. The reported endpoints are calibration-set agreement, count-based classification and electrical driver timing; accuracy on held-out data, physical compression-to-light/sound latency and educational effectiveness require separate evaluation.

## 1. Introduction

Ground-based CPR training prepares cabin crew and airport personnel to coordinate emergency actions. Aviation guidance addresses competency-based assessment and emergency medical equipment training [1,2], while Chinese consensus recommendations address CPR training [3]. Individual compression and ventilation records can complement instructors’ assessments by documenting the sequence and timing of actions.

Compression depth, rate, and recoil are central to CPR training. A 30:2 compression-to-ventilation sequence applies to the corresponding adult basic life support protocol [4]. Feedback should address specific educational objectives [5]. Previous studies have examined real-time feedback during training and resuscitation [6,7]. Smartphones, smartwatches, motion-capture systems, and interactive manikins record CPR actions through different measurement methods [8,9,10,11]. Automated compression devices serve a separate purpose by providing mechanical assistance [12].

Reviews and simulation studies link feedback to skill acquisition, retention and performance [13,14,15]. The present study addresses the measurement and event-detection stages that underpin such feedback through bench tests of a manikin-mounted prototype; educational outcomes require learner studies.

The controller processes continuous strain signals and binary airflow-switch transitions through separate paths, then associates compression features and ventilation events within a common training state [16,17]. Distinct stages for signal conversion, candidate-cycle detection, depth/recoil classification and feedback decisions allow stage-specific errors to be examined. The airflow channel supplies threshold-crossing events rather than tidal-volume measurements.

Building on Liu’s 2025 undergraduate prototype and initial bench records [18], this study quantifies how sensing and event decisions shape CPR feedback reliability. Laser-reference and repeated-loading tests characterise calibration agreement, hysteresis and installation dependence. Qualified-compression and invalid-action trials distinguish mechanical-cycle detection from training-quality decisions, while controlled airflow inputs and an electrical input-to-driver benchmark quantify trigger response and feedback timing. The contribution is an application-specific operating envelope that links each performance result to a defined measurement or decision stage. Calibration-set agreement and electrical driver timing are reported under their respective test definitions. Ground-based aviation training is the intended application; in-flight performance, clinical efficacy and learning outcomes require separate evaluation.

## 2. Materials and Methods

### 2.1. Prototype Architecture and Installation

The prototype is used with an adult CPR manikin (medical-teaching-equipment supplier, Chengdu, China) during ground-based training. *A* BF350-3AA strain gauge (Zemic Electronic Co., Ltd., Hanzhong, China) measures local deformation of an elastic chest component. The conversion to millimetres requires empirical calibration and depends on gauge location and installation of the tested manikin. We use equivalent depth to distinguish this estimate from an independently measured displacement. No patient physiological measurements were collected.

**Figure 1:**
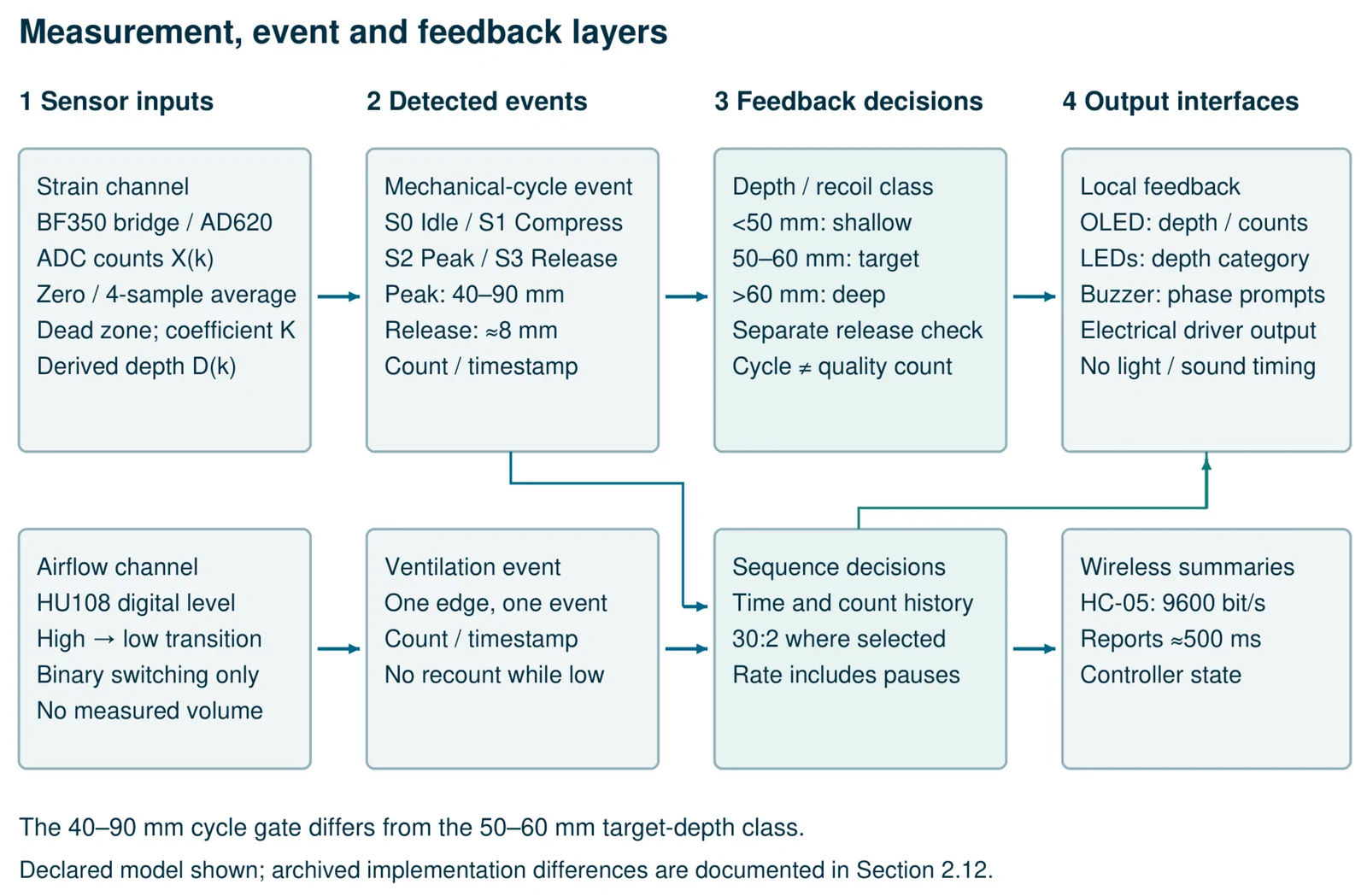
Separation of sensor inputs, detected events, feedback decisions and output interfaces. The strain path produces an equivalent-depth feature; the airflow path produces binary transitions. Mechanical-cycle and ventilation events provide counts and timing for training decisions. The 40–90 mm candidate-cycle window differs from the 50–60 mm target-depth class, and an airflow trigger does not establish delivered ventilation volume. Arrows represent the declared processing model rather than measured traces. Section 2.12 describes differences in the archived implementation.

The main controller is an STM32F103C8T6 (STMicroelectronics, Geneva, Switzerland). The strain signal passes through a bridge circuit, an AD620 amplifier (Analog Devices, Inc., Wilmington, MA, USA) and an analogue low-pass filter before 12 bit ADC acquisition. HU108 (electronic-components supplier, Wenzhou, China) supplies a digital switch level. An OLED, LEDs and a buzzer provide local feedback, while an HC-05 module (Guangzhou HC Information Technology Co., Ltd., Guangzhou, China) transmits wireless summaries. The prototype uses a 5 V USB supply and a nominal processing interval of approximately 5 ms. The declared Bluetooth configuration is 9600 bit/s, with one summary approximately every 500 ms. Figure 1 distinguishes sensor inputs, derived features, detected events, feedback decisions and output interfaces. Table 1 lists the principal operating parameters. Supplementary Tables S1–S22, Figures S1–S4 and Notes S21–S22 provide the supporting records in a separate Supplementary Materials file.

**Figure 2:**
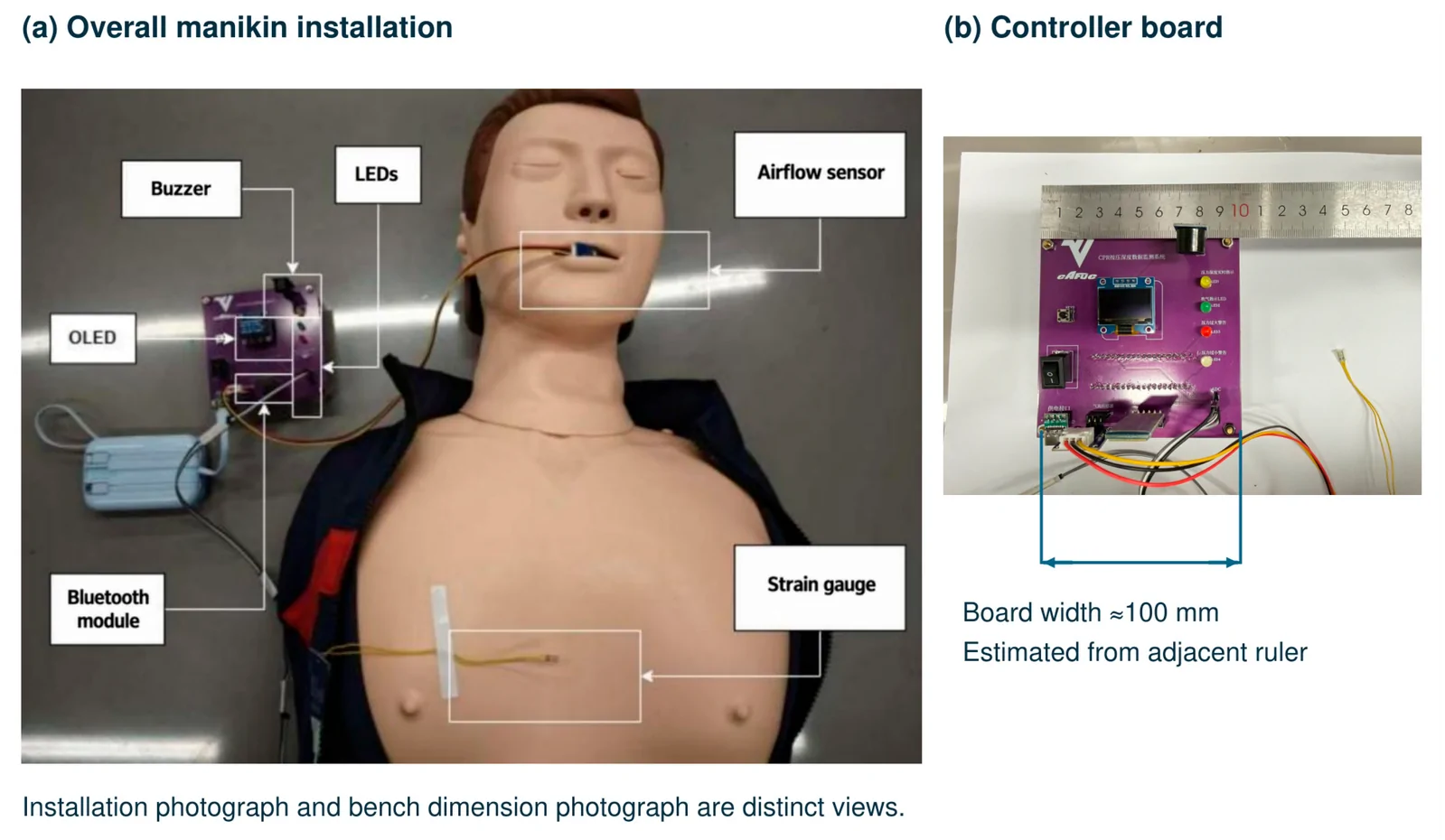
Overall installation and controller-board dimension. (**a**) Annotated manikin setup showing the sensing and feedback components. (**b**) Separate bench photograph with an adjacent ruler; the indicated board width is approximately 100 mm. The dimension is a photographic ruler estimate.

**Figure 3:**
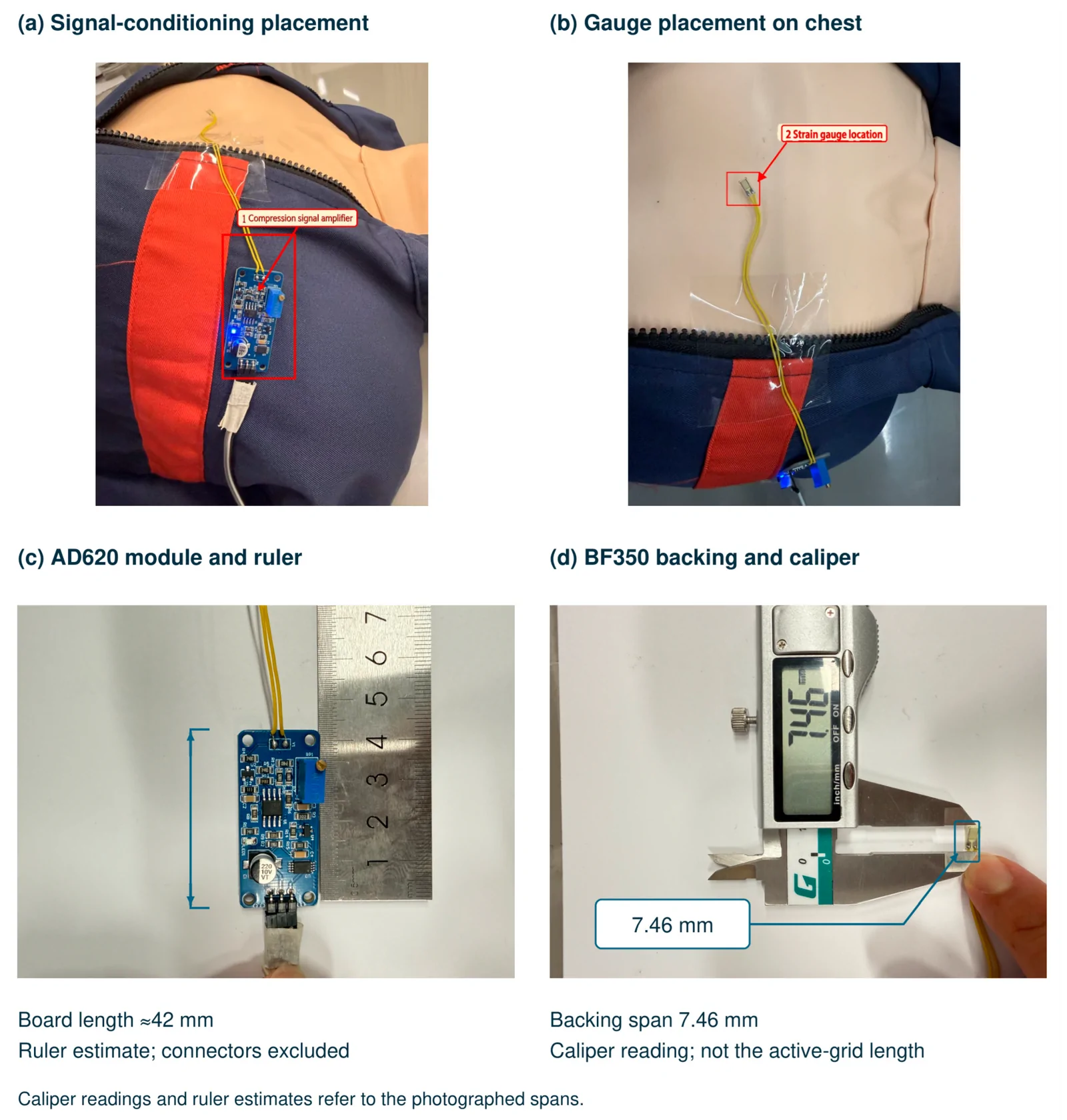
Strain-sensing installation and component dimensions. (**a**) Signal-conditioning module and wiring on the manikin. (**b**) Strain-gauge position on the chest. (**c**) AD620 board beside a ruler, giving an approximate 42 mm board length with connectors excluded. (**d**) Calliper photograph showing a 7.46 mm backing span. This span is distinct from the active resistive-grid length.

**Figure 4:**
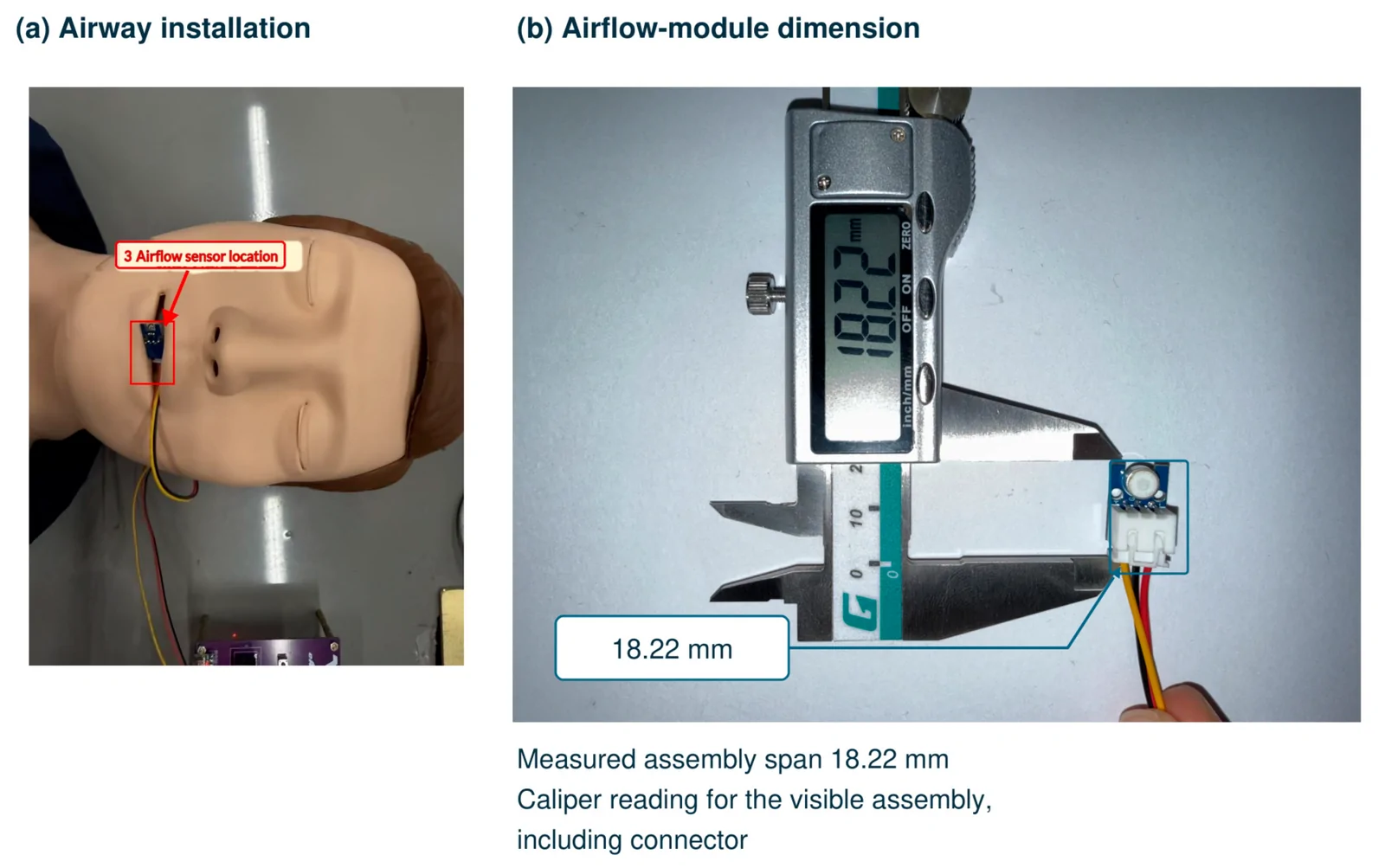
Airflow-switch installation and assembly dimension. (**a**) Module location near the manikin airway. (**b**) Separate calliper photograph displaying an 18.22 mm span for the visible module assembly, including its connector. The number describes the measured span rather than a sensing diameter or mounting clearance.

The main board is positioned beside the manikin, with the BF350 bonded to a loaded chest region and HU108 near the airway. Figure 2, Figure 3 and Figure 4 retain these installation views and add component photographs with visible measuring tools. The controller-board width is approximately 100 mm and the AD620-board length approximately 42 mm, estimated from adjacent rulers. The calliper photographs show a 7.46 mm strain-gauge backing span and an 18.22 mm airflow-assembly span, including its connector. These values describe the photographed spans, with ruler estimates distinguished from calliper readings. Signal wiring is routed away from LED and buzzer switching returns where possible.

The unloaded zero level is acquired before each session. During operation, the display reports equivalent depth and confirmed mechanical-cycle counts. Airflow falling edges update the ventilation count and phase prompts. The processing chain separates sensor measurements, detected events, and feedback decisions.

### 2.2. Strain Conditioning and Equivalent Depth

Compression deforms an elastic component inside the manikin, producing a differential bridge voltage through the BF350 resistance change. An approximately 248 Ω resistor sets the AD620 gain to approximately 200. An analogue low-pass filter precedes ADC acquisition. The hardware and protocol tables identify the baseline-acquisition and depth-conversion settings.

At startup, an unloaded baseline is acquired from 32 ADC readings. Equation (1) defines an ideal four-sample temporal moving average. At uniform 5 ms intervals, the samples span 15 ms and give a 7.5 ms group delay. The archived source instead averages four successive ADC conversions within one call and applies a 5 ms delay after other loop tasks. The calculated frequency response therefore applies to the ideal temporal model, with the archived implementation distinguished in Section 2.12.

(1) $$\bar{X}(k)=\frac{1}{4}\sum_{i=0}^{3}X\left(k-i\right)$$

Recorded peak-to-peak ADC fluctuations at rest were approximately 65–80 counts. To suppress small unloaded disturbances, Δ*X*(*k*) = $\left|\bar{X}(k)-X_{0}\right|$ is defined, with a dead zone Δ_0_ of 90 counts. Beyond this dead zone, the empirical coefficient *K* converts the signal to equivalent depth using Equation (2). The original settings used *K* of approximately 0.1 mm/count and a 90-count dead zone. The conversion was recalibrated for the revised static comparison; the revised ADC-domain parameters were not retained in the supplied summary records. These signal-domain parameters cannot be uniquely recovered from the retained depth pairs alone.

(2) $$D(k)=K\cdot \max\{0,|\bar{X}(k)-X_{0}|-\Delta_{0}\}$$

For the original static session, the author reports *X*_0_ = 1245 ADC counts from 32 unloaded readings at 24 ± 1 °C. Supplementary Table S22 records these declared settings and distinguishes them from the inspected firmware. *K* = 0.1000 mm/count is the adopted nominal coefficient, not the exact constrained least-squares optimum for the retained five points. Baseline, dead zone and scale remain fixed only within the declared session model; their operating-time and environmental stability is unverified (Table S21). These historical settings do not establish the revised laser-fit parameters.

The coefficient *K* in Equation (2) approximates the strain-to-depth relation over an installation-specific calibrated range. Placement and reinstallation tests refitted *K* for each configuration, quantifying performance after recalibration. Residual errors and loading–unloading separation characterise departures from the linear model; transfer of a fixed coefficient is a separate evaluation.

The original recorded ADC magnitudes *A* = |$\bar{X}$ − *X*_0_| were 278, 468, 675, 816 and 1038 counts. With *K* = 0.1000 mm/count and Δ_0_ = 90 counts, Equation (2) reproduces the original estimates (Table S9). Above the dead zone, *D* = *K*(*A* − Δ_0_), so the subtraction remains an offset. A diagnostic least-squares fit with zero and dead zone fixed gives *K* = 0.10222 mm/count; 0.1000 is therefore the recorded conversion setting rather than the exact minimiser for these pairs. These original settings do not determine the recalibrated parameters of the revised laser-reference comparison.

The revised static comparison uses directly measured laser displacement as the reference at five loading masses. The same five points were used for recalibration and assessment. At loading point *i*, ${\hat{D}}_{i}$ denotes estimated depth, *D_i_* denotes laser-reference depth, and *e_i_* is their signed difference, as defined in Equation (3). Positive values indicate overestimation and negative values indicate underestimation. Mean bias, mean absolute error (MAE) and root mean square error (RMSE) are calculated from the five signed system-minus-laser differences using Equation (4). These metrics describe bias direction, average error magnitude and sensitivity to larger errors, respectively.

(3) $$e_{i}={\hat{D}}_{i}-D_{i}$$

$$\mathrm{Bias}=\frac{\sum_{i=1}^{n}e_{i}}{n}$$

$$\mathrm{MAE}=\frac{\sum_{i=1}^{n}\left|e_{i}\right|}{n}$$

(4) $$\mathrm{RMSE}=\sqrt{\frac{\sum_{i=1}^{n}{e_{i}}^{2}}{n}}$$

### 2.3. Mechanical Cycle and Airflow Event Detection

The algorithm is represented by four logical stages, S0 idle, S1 compression, S2 peak accepted and S3 release confirmed. The declared cycle gate accepts peaks within 40–90 mm and confirms release near 8 mm. These logical stages distinguish initiation, acceptance and completion. The archived C implementation uses Boolean cycle-active and cycle-valid flags and accepts any in-cycle sample within 40–90 mm; it is not an exact implementation of peak-only acceptance or a 50–60 mm qualified-compression counter. Section 2.12 records this version distinction.

**Figure 5:**
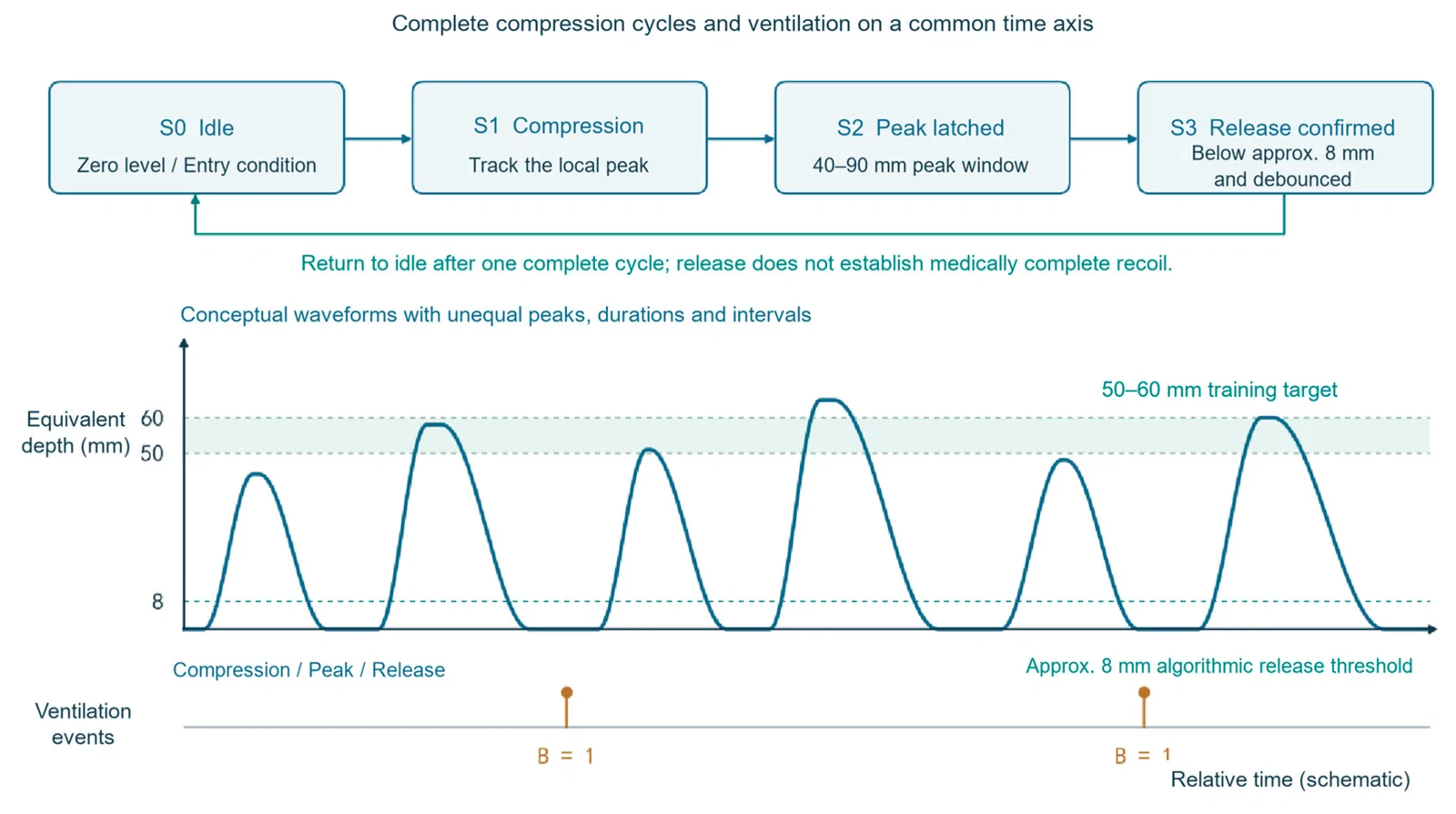
Conceptual compression states and ventilation events on a shared time axis. The waveforms illustrate state transitions rather than measured traces. The shaded band marks the 50–60 mm training target. Equivalent depth is not compression force, and the release threshold is not a clinical criterion for complete recoil.

The 40–90 mm candidate-cycle window admits shallow 40–50 mm and deep 60–90 mm peaks into sequence processing. Quality classification uses separate depth classes, <50 mm, 50–60 mm inclusive, and >60 mm. Peaks outside the candidate window are excluded from confirmed-cycle counting. The window is an engineering setting without comparative optimisation, and the approximately 8 mm release threshold marks an algorithmic cycle boundary rather than medically complete recoil. Figure 5 illustrates the state sequence.

For the additional classification evaluation, a qualified compression was defined by the laser reference as reaching 50–60 mm and fully releasing under the test protocol. A candidate cycle accepted within the broader 40–90 mm state-machine window is a distinct event. Counts reported as qualified compressions in the new tests are assessed against the narrower reference definition; candidate-cycle detection is not treated as proof of qualification.

For *N* confirmed cycles, with *N* ≥ 2, Equation (5) calculates the segment-average rate from the first and last confirmation times. Times *t*_1_ and *t_N_* are in seconds; the factor of 60 converts the result to compressions/min. Any ventilation pause between these confirmations remains in the observation interval.

(5) $$f=60\cdot \frac{N-1}{t_{N}-t_{1}}$$

Compression-only cadence is a separate boutwise quantity. For annotated continuous bouts *j* containing *N*ⱼ ≥ 2 confirmations, it can be computed as 60 Σⱼ(*N*ⱼ − 1)/Σⱼ(tlast,ⱼ − tfirst,ⱼ), excluding inter-bout intervals. No timestamp-based estimate of active cadence was available for reanalysis. The 100–120 compressions/min target applies to active cadence and should not be interpreted as an equivalent target for a rate diluted by ventilation pauses.

HU108 is high without airflow and switches to low when the trigger condition is met. Each high-to-low transition contributes one ventilation event, while a sustained low level contributes no additional counts. After the signal returns high, the controller waits for the next falling edge. The event therefore marks a threshold crossing rather than a measured flow magnitude.

Equation (6) defines the event-state vector from equivalent depth *D*(*k*), cycle state *S*(*k*), airflow-edge flag *B*(*k*) and controller time *T*(*k*). Depth and cycle state represent compression magnitude and cycle boundaries. The airflow flag and time associate ventilation triggers with the training sequence. This rule-based representation links features and events without assigning numerical fusion weights to strain values and switch levels.

***Z***(*k*) = [*D*(*k*), *S*(*k*), *B*(*k*), *T*(*k*)]^T^ (6)

### 2.4. Feedback and Wireless Summaries

The declared feedback design displays equivalent depth, cycle count, ventilation count and training time. Depth feedback distinguishes <50 mm, 50–60 mm inclusive and >60 mm, while the broader mechanical-cycle window serves sequence processing. Rate prompts based on Equation (5) describe the observed segment, including pauses, and require the interpretation specified above. The 30:2 sequence applies to the selected adult basic training protocol. These declared criteria (Table 2) must be distinguished from the different thresholds present in the archived firmware.

The declared HC-05 reporting configuration is 9600 bit/s with summaries approximately every 500 ms. Initial Bluetooth reception was checked for 60 s in line of sight. The archived project is supplied for source inspection, but its active entry point does not establish this reporting path or a complete timestamped sequence log.

The nominal 5 ms processing interval and 500 ms reporting interval describe separate scheduling stages. Section 3.4 reports a direct electrical input-to-driver benchmark alongside timing estimates from mixed sources. Physical compression-to-perceived-feedback latency is a distinct endpoint.

### 2.5. Bench and Functional Test Protocols

**Figure 6:**
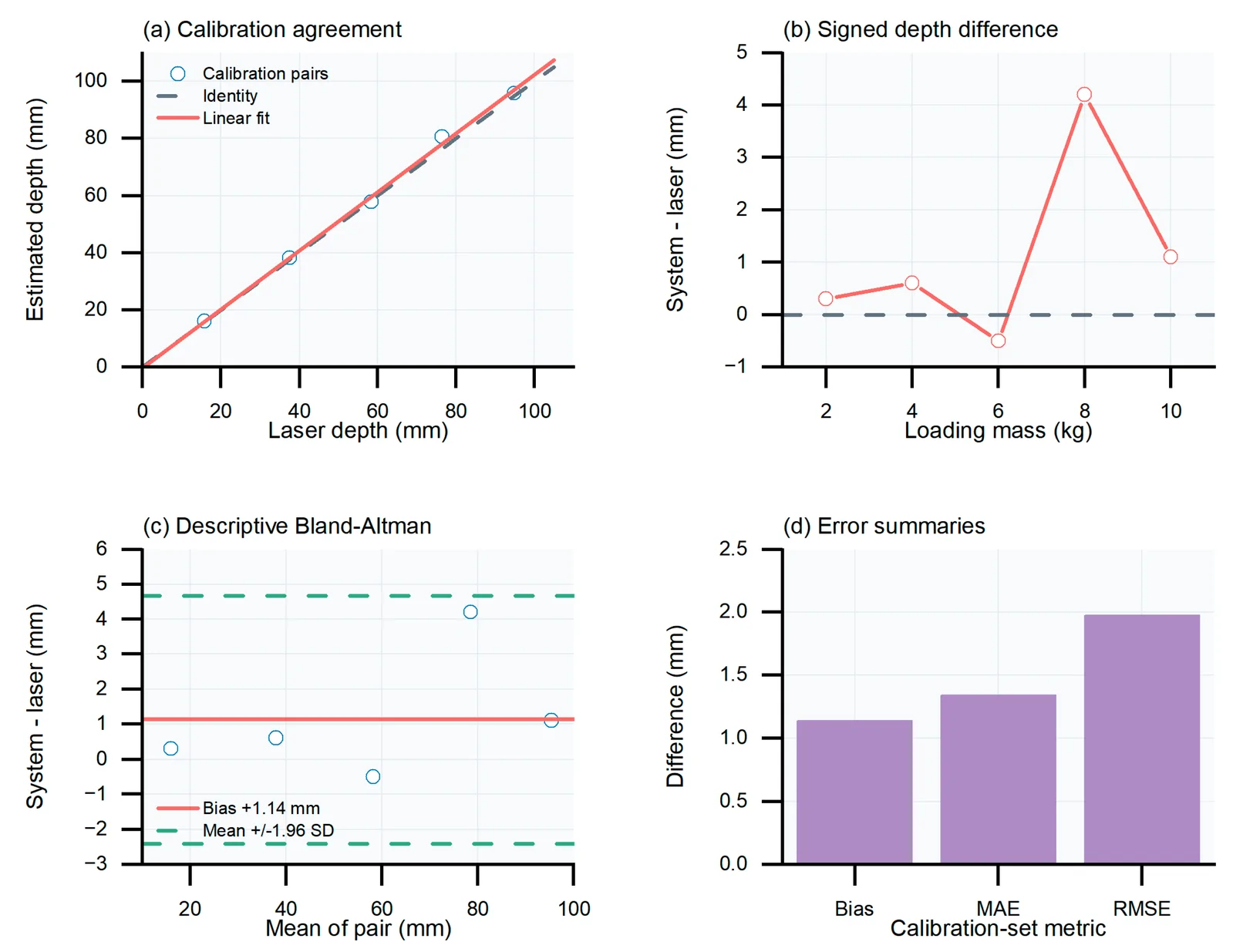
Laser-reference agreement for five pairs also used for recalibration. (**a**) Blue open circles indicate system/laser pairs, the grey dashed line is the identity line, and the red solid line is the linear fit. (**b**) Red open circles and their connecting line show signed system-minus-laser differences; the grey dashed line denotes zero difference. (**c**) Blue open circles show each pair’s difference versus its mean, the red solid line is the mean difference (+1.14 mm), and green dashed lines denote mean ±1.96 sample SD (−2.40 and +4.68 mm). (**d**) Purple bars show bias, MAE and RMSE on the calibration set. Connecting lines are visual guides; the agreement lines are exploratory rather than validated population limits.

**Figure 7:**
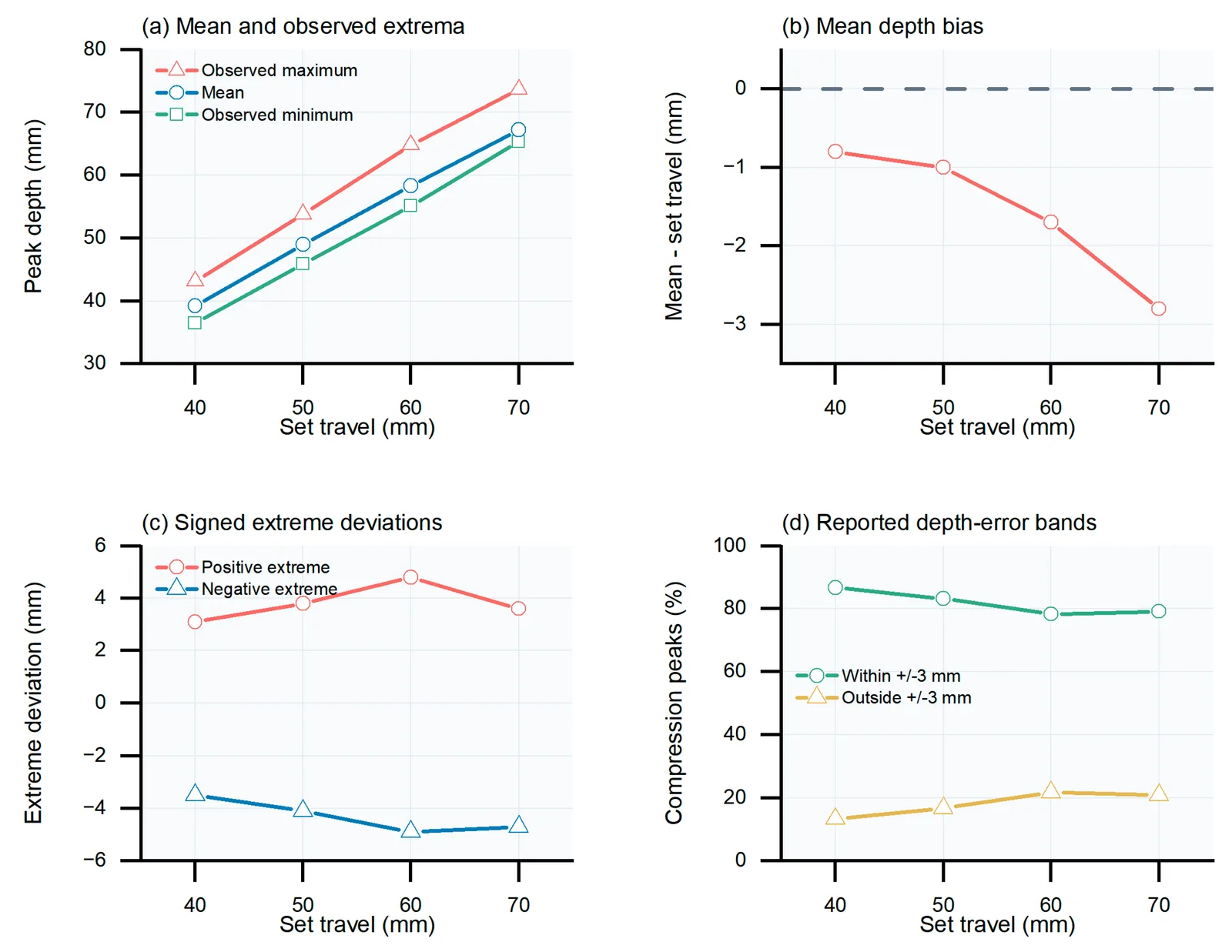
Archival dynamic summaries also documented in the precursor thesis [18]. (**a**) Blue open circles indicate mean peak depth, red open upward triangles the observed maximum and green open squares the observed minimum. (**b**) Red open circles show mean bias relative to set travel; the grey dashed line denotes zero bias. (**c**) Red open circles and blue open upward triangles show positive and negative extreme deviations, respectively. (**d**) Green open circles show proportions within ±3 mm and yellow open upward triangles proportions outside ±3 mm. Each condition summarises 120 cycles. Connecting lines are visual guides. Extrema are individual-cycle observations rather than SD, confidence intervals or paired laser agreement limits.

The initial dynamic and functional summaries overlap with Liu’s 2025 thesis [18] and are retained as archival prototype records, not independent replications. Revised Table 3 replaces the initial nominal-depth data with new laser-reference pairs. Table 4 and Table 5 retain their archival status, and Figure 6 and Figure 7 are redrawn from the respective released source tables. Supplementary Table S20 maps each result to its source and reproduction scope.

The revised static comparison used loading masses of 2, 4, 6, 8 and 10 kg and a calibrated laser sensor (PHOSKEY, Shenzhen, China) measuring displacement independently of the strain signal and manikin load–travel relation. One system/laser pair was retained per mass. The five pairs were used for both recalibration and assessment, defining a calibration-agreement test with a separate physical instrument. The released summaries lack the reference calibration certificate, full measurement geometry, raw ADC time series and revised ADC-domain conversion constants.

The original nominal depths were obtained from the manikin load–travel relation. No manufacturer calibration record or uncertainty specification for that relation was available. Calibrated loading mass establishes the force input but does not independently establish displacement traceability [19]. The revised Table 3 therefore uses directly measured laser displacement. The additional five load–reference-depth values in Table S1 follow *d*_ref_ = 9.620m−0.020 mm (*m* in kg), with R^2^ = 0.9999989. This is a descriptive fit to five rounded values over the tested 19.2–96.2 mm interval; it does not establish a universal reference curve or the accuracy of strain-derived depth.

Dynamic loading was performed at 100 compressions/min, with 120 cycles at each set travel of 40, 50, 60 and 70 mm. Each cycle included approximately 150 ms of compression, 50 ms of holding, 100 ms of recoil and 300 ms of relaxation. For each condition, the retained summary reports mean peak depth, extreme signed deviations and the proportion of peaks within ±3 mm of set travel. The four records describe 480 tested cycles; separately repeated sessions were not documented.

Original functional checks covered 20 brief button presses, ten routine breaths, weak-airflow triggering and Bluetooth reception. The Bluetooth check received approximately 120 frames over 60 s at approximately 10 m line of sight. The original weak-flow test lacked a denominator. Additional controlled-input airflow, electrical timing and laser-reference classification experiments are described in Section 2.11.

### 2.6. Error Definitions and Descriptive Analysis

Equation (4) summarises *n* = 5 retained calibration pairs, with the bias defined as system-estimated depth minus laser-reference displacement. Linear regression describes association between the two depths, while the difference-based metrics describe agreement. Mean ±1.96 sample-SD lines provide an exploratory agreement display for this five-point calibration sample [20,21].

Dynamic bias compares reported mean peak depth with set travel. The tabulated extrema yield an observed range, not an SD, confidence interval or agreement limit. The archival summaries contain no paired laser readings per cycle, so they cannot support a dynamic Bland–Altman analysis or reconstructed 50/60 mm classification errors. Outside-±3 mm percentages are complements of the rounded reported within-band percentages. No hypothesis tests or participant-level analyses were performed.

The accompanying package releases the available summaries, individual trial counts where retained, variable definitions and analysis code. It distinguishes source-transcribed observations from calculated fields and preserves conflicting source entries without treating them as extra measurements. Summary-level reproduction and firmware availability are described in Section 2.12.

### 2.7. Signal Chain Characterisation

Electronic-interface assessment distinguishes the sensor, analogue conditioning, digitisation and event-processing stages, following the system-level characterisation approach discussed by Laganà et al. [22]. Frequency response, noise, drift and timing are assigned to their respective stages. Component specifications [23], calculated filter properties and installed-chain measurements are reported separately; the ultrasonic performance values in [22] are specific to that sensing modality.

The ideal four-point moving-average response was calculated for a uniformly spaced nominal sampling rate of 200 Hz. Its group delay is half of the three sampling intervals between the oldest and newest samples. This gives a half-power frequency of 22.77 Hz, a first spectral null at 50 Hz, and 7.5 ms group delay away from the nulls. Calculated attenuation is 0.0149 dB at 100 compressions/min and 0.0214 dB at 120 compressions/min. These values characterise only the assumed digital filter, not measured BF350–AD620–ADC bandwidth or total latency.

The direct electrical benchmark supplies the main quantitative timing result. Engineering-derived five-rate budgets and earlier summaries with unverified endpoints are archived separately. Rate-specific CPR-sequence jitter requires paired physical-reference and output timestamps; analogue bandwidth, spectral noise and drift coefficients require acquisition records beyond the retained aggregates.

### 2.8. Loading–Unloading and Installation Tests

Loading and unloading summaries used laser-reference displacements of 19.2, 38.5, 57.7, 76.9 and 96.2 mm at 2–10 kg. Branch separation was unloading-minus-loading mean depth, normalised by reference depth for relative separation. Placement tests compared the centre with 20 mm leftward and upward offsets; a separate summary covered three independent installations. Each configuration was recalibrated, so the summaries characterise installation dependence after refitting. Raw branch cycles, calibration ADC records and a separate fitting/evaluation split were unavailable.

### 2.9. Algorithm Comparison and Summary Analysis

Author-supplied summaries compared the current and comparator algorithms at 40–70 mm travel, 80–120 compressions/min and selected boundary conditions. Reported endpoints included cycle accuracy, ventilation matching, phase errors and mean latency. Comparator source code, matched-input records, event denominators and common timing endpoints were not supplied. These comparisons are descriptive and cannot isolate the effect of shared-state integration from other algorithm differences. The meaning of the 15 mm recoil setting in the boundary summary was not resolved.

Analysis was descriptive. Installation-coefficient relative standard deviation was 100 times sample SD/mean, using three installation values and the *n* − 1 SD denominator. Condition means were not pooled into event-level accuracy, and no inferential tests or uncertainty intervals were reconstructed. Boundary ventilation percentages below 100% despite zero misses were not used as reconciled detection rates. These entries and undefined ± rate errors are retained with notes in Tables S5 and S6. Source summaries are provided in Tables S1–S6.

### 2.10. Laser-Reference Tests of Qualified-Compression Classification

Seven scenarios were tested for 60 s each, with ten repeated trials per scenario on the same installation. Laser displacement supplied the depth and recoil reference. Valid full-release compressions at 60, 80 and 120 compressions/min had nominal reference counts of 60, 80 and 120 per trial. An irregular 60–120 compressions/min condition had a reported mean of 90 valid reference compressions per trial; individual trial reference counts were not supplied. Shallow compressions (<50 mm, full release) and incomplete-release compressions (target depth reached, but recoil did not return below the 8 mm threshold) each contained 60 invalid actions per trial. A 60 s resting condition had no prescribed action opportunities. The supplied per-trial files report true-positive counts for the four valid conditions and false-positive counts for the three invalid/resting conditions. Here the output endpoint is the count labelled as qualified, rather than all mechanical candidate cycles.

For fixed-count valid conditions, FN was reference count minus the reported TP count, and sensitivity was TP/(TP + FN). The irregular-rhythm ratio used mean TP divided by the reported mean reference count, without reconstructing individual FN values. For shallow and incomplete-release conditions, FP/60 and 1−FP/60 are reference-action-normalised count estimates under a one-output-per-action assumption. Resting false counts are reported per minute. Mean ± sample SD uses ten technical repeats on one installation. Event-matching tolerance, synchronised reference/output timestamps, individual irregular reference counts and the generating qualified-counter firmware were not available, which limits event-wise interpretation.

### 2.11. Additional Controlled Airflow Timing and Classification Tests

Controlled forward-inspiratory pulses were delivered with a calibrated 1 L syringe (Shanghai Glass Instrument Factory, Shanghai, China) (stated volume accuracy ±0.5%) through a reference flowmeter (Wright Respiratory Products Ltd., London, UK) (0–120 L/min, stated accuracy ±2%) and HU108 in series. Reference flow and switch level had stated 1 ms alignment. We applied 30 pulses at each of 500, 300, 200 and 100 mL, with approximate peak flows of 30, 18, 12 and 6 L/min. Pulse detection was detected events divided by 30. Ten rising-flow tests summarised the switch-on threshold. Three 5 min no-flow blocks yielded 900 non-overlapping 1 s windows; specificity was TN/(TN + FP), and false edges were also expressed per minute. Volume and peak flow covaried. Sequential windows were not independent installations; reverse flow, leakage and waveform distortion were not tested.

A differential electrical step from a function generator (RIGOL Technologies Co., Ltd., Suzhou, China) was injected directly at the AD620 input. A 100 MHz oscilloscope (RIGOL Technologies Co., Ltd., Suzhou, China) compared the 50% point of the input rising edge with the 50% transition of the LED-driving GPIO for 100 triggers. The stated instrument time resolution was 1 ns; this is not a complete timing-uncertainty estimate. A 1 GHz logic analyser and firmware markers were used for scheduling and state-transition checks. The electrical benchmark bypasses the strain bridge and manikin mechanics, and its endpoint is a driver signal rather than measured light or sound onset. The additional rate summary is an engineering-derived envelope for 60–140 compressions/min. The stated 120 cycles per rate describe the workload context, not 120 supplied synchronised latency pairs. Its stage budget combines measured marker intervals, datasheet/circuit estimates and engineering allowances. Sums of stage extrema are not an observed end-to-end jitter distribution. Tables S12–S15 distinguish the reported budget from the direct electrical benchmark.

A separate depth-classification summary covered 120 cycles at each of 40, 50, 60 and 70 mm set travel, with a stated laser sampling rate 1 kHz, accuracy ±0.1 mm and time alignment 1 ms. *K* and the dead zone were fitted using these same data. Matrix rows denote predicted class and column reference class; exact 50 and 60 mm values belong to the target class. Only the internally consistent 40 and 50 mm matrices are used as classification results. Unreconciled higher-depth matrices, paired means and spans are archived in Tables S16b and S18, and the original distribution-based CSV is retained as a model estimate in Table S14.

### 2.12. Provenance and Reproducibility

The historical static records differ at 8 kg. Table 5.1 in Liu’s thesis [18] reports 74.6 mm, whereas the submitted nominal-depth comparison reports 72.6 mm, both against 76.9 mm. The retained 816-count magnitude reproduces 72.6 mm through Equation (2); the thesis ADC and zero records are unavailable, so the 2.0 mm difference cannot be assigned to a specific cause. Supplementary Note S21 provides the batch clarification and source-document inconsistencies.

The analysis programme reproduces numerical Table 3, Table 4 and Table 5 and the source values for Figure 6 and Figure 7; the Origin project supplies the revised figure presentation. The archived Keil project uses four within-call ADC conversions, integer depth mapping with a 90-count dead zone, 40–90 mm in-cycle acceptance and an 8 mm release threshold. The release supports summary-level numerical reproduction and source inspection. Full experimental replay requires version-linked generating firmware, including the separate qualified-compression counter and matched comparator, together with the unretained continuous acquisition and per-event reference/output records.

## 3. Results

### 3.1. Static Depth Estimates

Across five laser-reference calibration pairs spanning 15.8–94.8 mm, mean system-minus-laser bias was +1.14 mm, MAE 1.34 mm and RMSE 1.98 mm (Table 3 and Figure 6). The fitted relationship was estimated depth = 1.02594 × laser-reference depth − 0.32804 mm, with R^2^ = 0.99745. The descriptive Bland–Altman display has mean bias +1.14 mm and mean ±1.96 sample-SD lines at −2.40 and +4.68 mm. These metrics quantify agreement after recalibration on the same five pairs; the agreement lines are exploratory.

The largest absolute difference occurred at 8 kg, with an estimated depth of 80.6 mm and laser-reference depth of 76.4 mm. The +4.2 mm bias corresponds to 5.50% overestimation relative to the laser reference. At each remaining load, the absolute difference was at most 1.1 mm.

### 3.2. Dynamic Peak Deviations

Mean peak-depth biases were −0.8, −1.0, −1.7 and −2.8 mm at 40, 50, 60 and 70 mm set travel, respectively (Table 4 and Figure 7). The proportion within ±3 mm was 78.3–86.7%, and the largest observed absolute single-cycle deviation was 4.9 mm.

Observed peak-depth spans were 6.6, 7.9, 9.7 and 8.3 mm across the four conditions. Mean estimates at 50 and 60 mm set travel were 49.0 and 58.3 mm, respectively. The within ±3 mm endpoint measures proximity to set travel; target-depth compliance requires classification against the separate 50–60 mm range.

### 3.3. Functional Checks

Archival functional checks recorded 20/20 button responses, 10/10 routine-airflow triggers and one missed weak-flow trigger with an undocumented denominator (Table 5). Controlled 30-pulse conditions and a defined no-flow exposure are reported separately in Section 3.8. Approximately 120 Bluetooth frames were received over 60 s without visibly corrupted characters, documenting reception cadence; packet-loss probability and one-way latency require additional measurements.

### 3.4. Electrical Feedback Timing and Signal Chain Limits

Table 6 reports the direct electrical benchmark alongside the engineering timing budget. Earlier six-rate summaries remain in the provenance archive because their input/output endpoints are unverified. The electrical result and engineering budget retain separate measurement definitions.

Across 100 electrical step tests, input-to-GPIO delay had a mean of 26.4 ms, minimum of 19.7 ms, maximum of 37.2 ms and observed range of 17.5 ms (Table 6 and Table S15). Corresponding budget entries were 26.0, 19.3 and 36.7 ms, giving relative differences of +1.54%, +2.07% and +1.36%. The observed maximum exceeded the budget by 0.5 ms, placing it outside the proposed budget envelope. The measured endpoint spans electrical input-to-driver output and excludes manikin mechanics, strain-bridge response and optical/acoustic onset.

The five-rate engineering series allocates peak-delay means of 25.2–27.1 ms and count-confirmation means of 32.2–34.1 ms (Table S12 and Figure S4). At 100 cpm, the corrected release allowance is 7 ms mean and 5–10 ms range, giving a count budget of 33 ms mean and 24.3–46.7 ms range. Adding the 0–500 ms send schedule and 18–22 ms transport allowance gives a Bluetooth budget of 303 ms mean and 42.3–568.7 ms range (Table S13). These are calculations, not observed end-to-end jitter. The 10 ms processing allowance is distinct from the ideal 7.5 ms temporal-filter group delay, which the archived burst-average code does not establish.

Resting fluctuations of 65–80 ADC counts peak to peak provide the available system-level noise observation. RMS noise, noise density, SNR and ENOB require the acquisition duration, bandwidth, raw records and voltage reference. Supplementary frequency-response and drift summaries retain unresolved transfer-function or unit inconsistencies and are excluded from measured chain-cutoff and drift-coefficient estimates.

### 3.5. Loading–Unloading Behaviour and Calibration Variability

**Figure 8:**
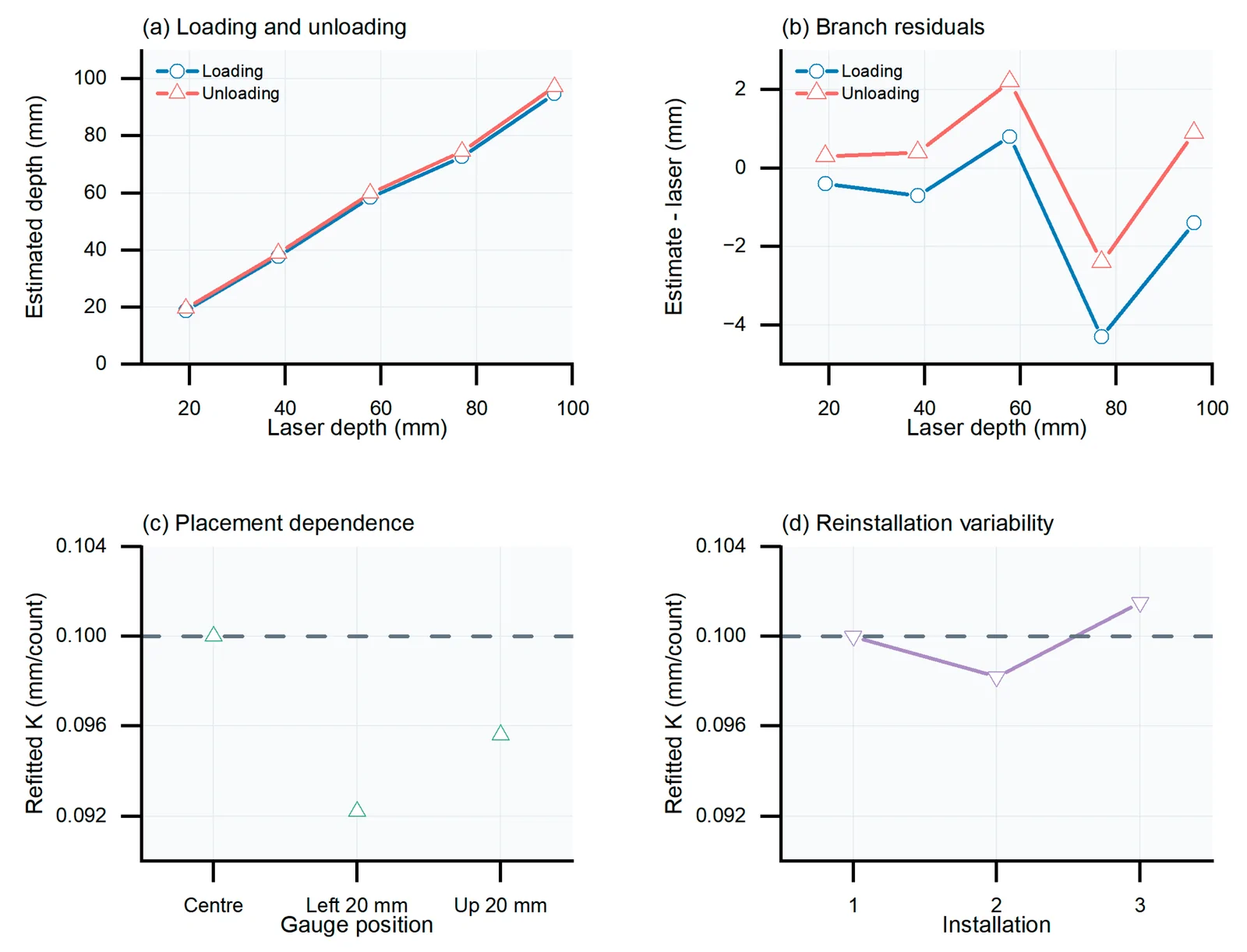
Loading-direction and installation dependence. (**a**,**b**) Blue open circles indicate loading and red open upward triangles unloading, showing mean estimates and signed residuals against the listed laser-reference depths, respectively. (**c**) Green open upward triangles show separately refitted *K* at the centre and the 20 mm leftward and upward gauge offsets. (**d**) Purple open downward triangles show separately refitted *K* in three installations. Grey dashed horizontal lines in (**c**,**d**) denote the initial 0.1000 mm/count setting. Connecting lines are visual guides. The branch summaries lack underlying repeat-level records. Source data are Tables S1–S3.

Across five laser-reference depths, unloading-minus-loading separation increased from 0.7 to 2.3 mm, with an unweighted mean of 1.48 mm (Figure 8a and Table S1). Loading errors ranged from −4.3 to +0.8 mm, and unloading errors from −2.4 to +2.2 mm (Figure 8b). At 8 kg, the loading estimate was 72.6 mm against the 76.9 mm reference displacement, giving a −4.3 mm (−5.59%) error. The original recorded ADC magnitude of 816 counts gives 0.1000 × (816 − 90) = 72.6 mm. The same model would require 859 counts for 76.9 mm, so the residual corresponds to a 43-count deficit. This establishes numerical consistency of the conversion, without uniquely identifying a mechanical cause. Unloading gave 74.5 mm, leaving −2.4 mm residual despite the 1.9 mm branch change. These values are distinct from the revised Table 3 pair, 80.6 mm estimated versus 76.4 mm laser depth, whose signed error is +4.2 mm.

Refitted *K* values were 0.1000, 0.0922 and 0.0956 mm/count at the centre, left-offset and upward-offset positions, respectively, corresponding to changes of −7.8% and −4.4% (Figure 8c and Table S2). After position-specific recalibration, reported static MAE was 1.52–2.14 mm and RMSE 2.09–2.76 mm. Across three installations, *K* was 0.0982–0.1015 mm/count, with mean 0.0999 and sample relative SD 1.65% (Figure 8d). Zero levels were 1231–1262 counts and reported RMSE 2.09–2.25 mm (Table S3). These results quantify between-installation calibration variability and support recalibration after mounting changes; operating-time drift and transfer of a fixed *K* are separate endpoints.

### 3.6. Descriptive Algorithm Comparison

**Figure 9:**
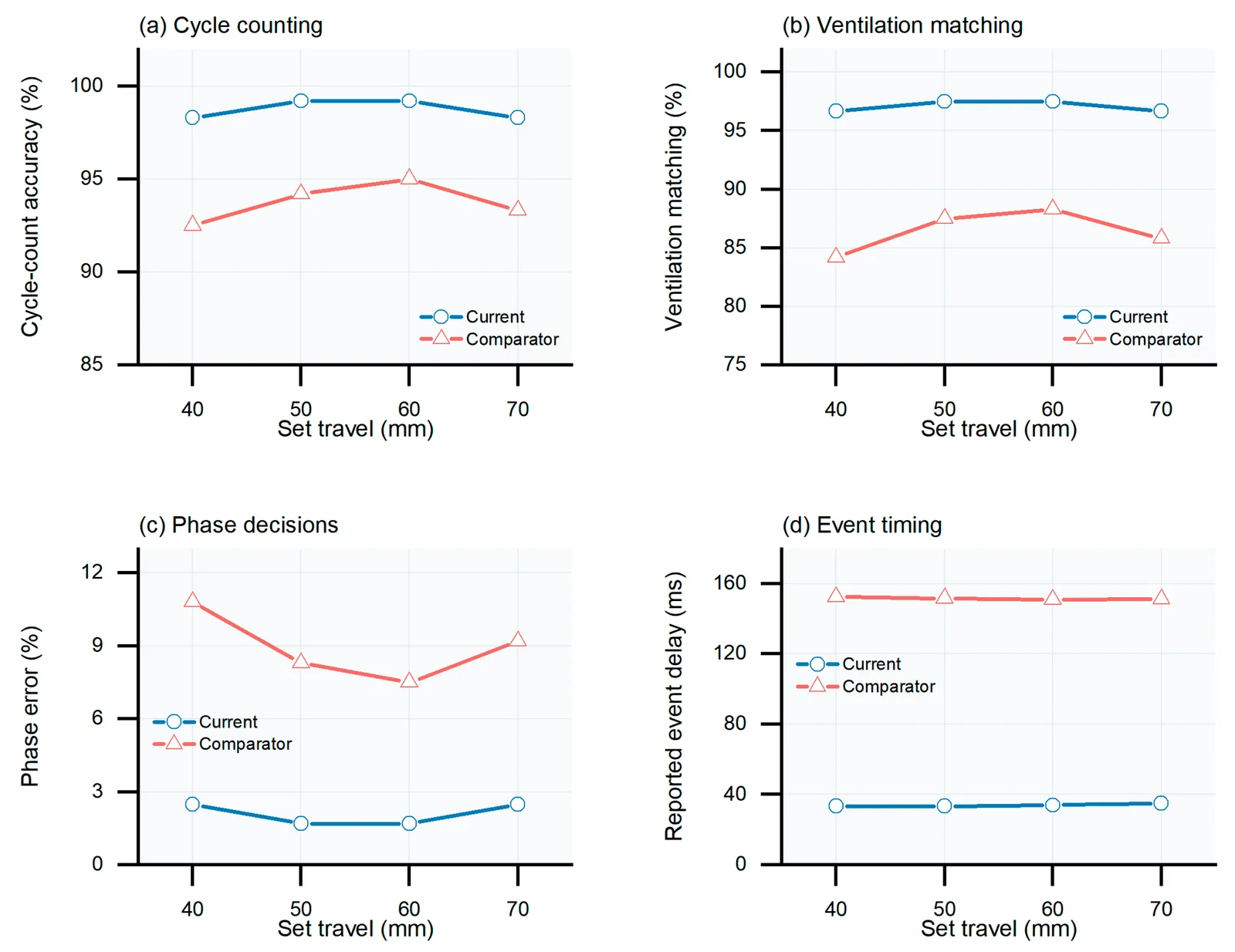
Descriptive current/comparator summaries at four set-travel conditions. Blue open circles and solid lines indicate the current algorithm; red open upward triangles and solid lines indicate the comparator. Panels show (**a**) reported cycle-count accuracy, (**b**) ventilation-event matching, (**c**) phase-prompt error and (**d**) reported mean event delay. Source summaries are in Table S4; connecting lines are visual guides. These supplied condition-level comparisons require comparator code, event denominators and verified common timing endpoints to attribute differences to channel integration. No commercial-device benchmark was performed.

At 40–70 mm set travel, the current algorithm had reported cycle-counting accuracy of 98.3–99.2%, compared with 92.5–95.0% for the comparator, a difference of 4.2–5.8 percentage points (Figure 9 and Table S4). Ventilation-event matching was 96.7–97.5% versus 84.2–88.3%, a difference of 9.2–12.5 percentage points. Phase-prompt error was 1.7–2.5% versus 7.5–10.8%. Reported mean event latency was 33.5–35.0 ms versus 150.9–152.3 ms, a condition-specific reduction of 116.2–118.8 ms. These timing differences remain conditional on confirmation of identical timing endpoints and measurement procedures.

Across 80, 100 and 120 compressions/min, reported cycle accuracy was higher by 5.0–6.7 percentage points and ventilation matching by 10.0–14.1 percentage points (Table S5 and Figure S1). Under reduced airflow, shallow compression and incomplete recoil, cycle-accuracy differences were 5.9, 14.2 and 14.2 percentage points; corresponding event-timing error reductions were 10.0, 9.2 and 8.3 percentage points (Table S6). These condition-level comparisons do not isolate the contribution of integration from differences in filtering, thresholds, detection rules or buffering.

### 3.7. Qualified-Compression Performance Against Laser Reference

At 60, 80 and 120 compressions/min, mean reported true-positive counts were 54.7, 74.8 and 105.6 per trial against 60, 80 and 120 reference actions, giving sensitivities 91.2%, 93.5% and 88.0% and mean false-negative counts 5.3, 5.2 and 14.4 (Table 7). At irregular 60–120 compressions/min, mean TP was 79.7 and mean reference count 90, yielding an aggregate ratio of 88.6% and a difference of 10.3 counts/trial. The latter is not a mean of ten per-trial sensitivities, because individual irregular reference counts were unavailable.

Invalid-action trials quantified false qualified-compression outputs (Table 8). Shallow actions produced 27.9 ± 1.73 false-positive counts per 60 reference actions (46.5%); incomplete release produced 12.3 ± 2.21 (20.5%). At rest, false outputs occurred in all ten trials, at 7.4 ± 1.71/min over 60 s (range 5–11). Resting seconds do not define negative event opportunities, so specificity and event-level false-positive probability are undefined for that condition. The results identify rejection of shallow actions, incomplete release and resting disturbances as priorities for the qualification logic.

Supplementary Tables S7 and S8 and Figure S2 provide individual trial counts and their distributions. The reported percentages summarise labelled counts relative to the laser-defined sequence. Event-matched confusion matrices require synchronised reference/output records and a matching tolerance, which were absent from the supplied files.

### 3.8. Controlled HU108 Trigger Response

The switch-on threshold summary was 12.5 L/min with CV 4.2% across ten tests. Pulse detections were 30/30, 27/30, 20/30 and 3/30 across the four decreasing volume/peak-flow settings, corresponding to 100%, 90.0%, 66.7% and 10.0% detection (Table 9 and Figure S3a). Misses were 0, 3, 10 and 27; no extra trigger was reported in those positive-input blocks. The three no-flow blocks contained 0, 1 and 0 false edges. The event occurred 192 s into block 2 and lasted approximately 120 ms. Across 15 min, this gives 0.067 false events/min. With 899 negative and one positive no-flow windows, specificity was 899/900 = 99.89%. These endpoints describe the tested pulse and window definitions rather than a universal threshold or long-term reliability.

### 3.9. Laser-Reference Depth Classification

The internally consistent calibration-set matrices gave 0/120 wrong classes at 40 mm and 14/120 (11.7%) at 50 mm (Table 10 and Figure S3b). In the 50 mm group, 11 reference-shallow cycles were predicted as target and three reference-target cycles as shallow, yielding the count-based target sensitivity of 54/57 = 94.7% and specificity of 52/63 = 82.5%. The same data fitted *K* and the dead zone. The 60 and 70 mm matrices remain excluded from accepted rates and combined accuracy because they conflict with supplied totals or depth spans (Table S18).

## 4. Discussion

### 4.1. Interpretation of the Dual Channel Architecture

The event-state representation links strain-derived features and airflow edges to a common training sequence while keeping measurements, candidate cycles, quality classes and feedback decisions distinct. This separation makes calibration error, event detection and quality classification individually assessable. The hardware channels build on the precursor thesis [18]; the application contribution is their stage-specific characterisation for CPR feedback. The comparative summaries describe differences between the supplied algorithms, while a matched ablation and version-linked source are needed to attribute an advantage specifically to shared-state association.

Table 11 places the prototype alongside quantitative reports for smartphone and smartwatch CPR feedback systems [8,9]. Published depth agreement and rescuer effectiveness provide complementary context to the present five-point calibration errors, with each endpoint retained under its original definition. Motion capture addresses CPR movement assessment [10], and interactive teaching manikins combine compression and ventilation functions [11]. The present study focuses on how strain conversion, airflow triggering and event decisions affect the operating envelope; comparative accuracy, cost and educational outcomes require matched evaluations.

Bibbò et al. [24] evaluated learned EEG–EMG fusion for an exoskeleton using unimodal and fusion ablations. For the present rule-based CPR sequence, the corresponding test would retain both sensor channels and vary event-state association alone. This provides a methodological route for isolating the contribution of association logic, while the exoskeleton’s classification scores and inference times remain specific to its task.

### 4.2. Measurement and Feedback Boundaries

The independent laser instrument anchors depth estimates to a physical displacement reference. MAE of 1.34 mm and RMSE of 1.98 mm quantify agreement after fitting the same five pairs. Position and installation tests extend this characterisation to separately refitted configurations. The next accuracy test is a new paired laser dataset evaluated with conversion parameters fixed beforehand, including transfer of one unchanged *K* across installations.

The historical 8 kg residual identifies a local departure from the constant strain-to-depth mapping. Reference-depth slopes derived from recorded ADC magnitudes are 0.13617 mm/count over 6–8 kg and 0.08694 mm/count over 8–10 kg, compared with the adopted *K* of 0.1000 mm/count. Estimate-to-ADC slopes equal *K* by construction. These calculations locate the conversion discrepancy but do not distinguish chest stiffness, friction, creep or loading geometry. Supplementary Table S10 retains author-reported diagnostic magnitudes, including a 0.2 mm peak-to-peak fluctuation. Overlapping definitions, bidirectional fluctuation and an unreconciled 0.7 mm cycle magnitude versus the 1.9 mm branch separation in Table S1 prevent an additive bias decomposition. A 60 s hold reduces transient loading effects without confirming the cessation of creep.

The 50 mm calibration condition produced an 11.7% classification error, locating an important quality boundary for subsequent testing with fixed parameters. Higher-depth summaries require reconciliation before contributing to accuracy estimates. Predicted classes at 60 mm conflict with the estimated depth span, and the reference classes at 70 mm conflict with the laser minimum; these records remain available for audit. The 40–90 mm candidate-cycle window is an engineering setting whose optimisation requires a separate comparison.

Qualified-compression trials distinguish quality decisions from the earlier 40–90 mm mechanical-cycle endpoint. Regular-rhythm sensitivity was 88.0% at 120 compressions/min, while shallow and incomplete-release false-positive proportions were 46.5% and 20.5%; every resting trial also produced false outputs. These observations locate the main classification problem in acceptance of non-qualifying actions. Synchronised waveforms and event logs are needed to distinguish threshold, noise, timing and state-transition contributions and guide refinement of the qualification logic.

Controlled HU108 tests quantify how the binary trigger depends on the applied airflow input. Detection was 3/30 pulses at 100 mL and approximately 6 L/min, compared with 30/30 routine pulses. The switch-on summary was 12.5 L/min with 4.2% CV, and no-flow specificity was 99.89% across sequential 1 s windows in three five-minute blocks. These results characterise tested inputs and exposure rather than tidal-volume accuracy or a universal waveform-independent threshold. Leakage, orientation, pulse shape, switch-off hysteresis and longer-term reproducibility remain priorities for further characterisation.

Equation (5) reports a segment-average cycle rate that includes intervening ventilation pauses. Active compression cadence is the relevant endpoint for the 100–120 compressions/min target and can be obtained with the separate boutwise formula when event timestamps and pause boundaries are available. The supplied records lack those timestamps, and illustrative 92 and 100 compressions/min examples are not experimental results. The 8 mm release threshold defines an algorithmic cycle boundary rather than complete clinical recoil.

The direct benchmark measures electrical input-step-to-GPIO delay. Physical compression onset, strain transduction, emitted light/sound and application display require additional timing endpoints. Engineering envelopes describe stage budgets, frame cadence describes reporting frequency, and the four-tap calculation describes an ideal temporal filter; each retains its own interpretation. Physical-reference-to-feedback timing and installed-chain analogue frequency response, spectral noise and drift are the next system-level characterisation tasks [22].

### 4.3. Scope and Next Validation Steps

The placement, reinstallation, loading-direction, algorithm and laser-labelled action tests define complementary parts of the prototype’s operating envelope. Ten technical repeats on one installation characterise within-installation behaviour; separately refitted configurations quantify calibration dependence. Generalisation across installations requires fixed-parameter tests, and attribution to event association requires matched comparison inputs. Synchronised reference/output records, an explicit matching tolerance and generating firmware versions would make the event-level metrics reproducible.

The prototype provides an airflow-event channel and local feedback interfaces for manikin training. Table 11 supplies the quantitative literature context; a same-condition commercial-device comparison is needed to evaluate relative accuracy, cost and portability. Learner studies are the appropriate next step for testing educational outcomes in the intended aviation setting. Findings from other feedback systems [13,14,15] motivate that evaluation but do not establish this prototype’s educational effectiveness.

### 4.4. Relationship to the Precursor Study

The 2025 thesis [18] provides the sensing platform, baseline counting approach and overlapping initial bench records, with reuse identified at the result level. The added physical-reference and controlled-input tests quantify calibration dependence, quality-boundary errors, weak-flow detection and a defined electrical feedback delay. Their contribution is an operating envelope that connects signal acquisition, event decisions and feedback quality in a CPR training application. This stage-specific evidence directs subsequent calibration, algorithm and training validation.

## 5. Conclusions

This study characterises a dual-channel manikin CPR prototype from strain conversion and airflow events to quality decisions and electrical feedback. Laser-reference calibration yielded an MAE of 1.34 mm and RMSE of 1.98 mm, while loading and installation tests quantified conversion dependence. Qualified-compression trials identified shallow-action and incomplete-release acceptance as priorities for algorithm refinement. Controlled HU108 tests resolved input-dependent detection and 99.89% specificity in the defined no-flow windows; the electrical input-to-driver benchmark yielded a mean delay of 26.4 ms. Together, these results provide a stage-specific operating envelope and a concrete basis for fixed-calibration, event-matched and physical-feedback validation. Source tables and analysis code support numerical reproduction of the released summaries, with archival and new results distinguished.

## Figures and Tables

**Table 1:** Prototype modules and operating parameters.

Module	Device or Method	Recorded Parameter or Function
Controller	STM32F103C8T6	12-bit ADC; approx. 5 ms updates
Deformation sensing	BF350-3AA + AD620	248 Ω gain resistor; gain approx. 200
Ventilation sensing	HU108	Airflow-edge events
Local feedback	OLED/LEDs/buzzer	Numerical, category and state prompts
Wireless summaries	HC-05	9600 bit/s; approx. 500 ms/frame

**Table 2:** Feedback criteria and output definitions.

Feature	Criterion and Interpretation	Output
Equivalent depth	Categories: <50, 50–60 and >60 mm	Depth and LED category
Mechanical cycle	Peak 40–90 mm, followed by release below approx. 8 mm	Confirmed cycle
Segment-average cycle rate	Prototype target 100–120 cpm; averaging includes pauses	Displayed sequence rate; differs from active cadence
Ventilation event	Airflow falling edge; 30:2 where applicable	Event count and phase prompt
Training time	Approx. 5 ms updates; 500 ms summaries	Countdown and Bluetooth state

**Table 3:** Revised static depth comparison against a calibrated laser displacement sensor. Bias is system-estimated depth minus laser-reference depth. Each row represents one retained pair; the same five points were used for recalibration and assessment.

Loading Mass (kg)	Laser-Reference Depth (mm)	Estimated Depth (mm)	Bias (mm)
2	15.8	16.1	+0.3
4	37.6	38.2	+0.6
6	58.4	57.9	−0.5
8	76.4	80.6	+4.2
10	94.8	95.9	+1.1

**Table 4:** Archival dynamic loading summaries at 100 compressions/min, also documented in the precursor thesis [18]. Each row represents 120 reported cycles. Extreme deviations are individual-cycle observations relative to set travel, not uncertainty intervals.

Set Travel (mm)	Mean Peak Depth (mm)	Maximum Positive Bias (mm)	Maximum Negative Bias (mm)	Within ±3 mm (%)
40	39.2	+3.1	−3.5	86.7
50	49.0	+3.8	−4.1	83.3
60	58.3	+4.8	−4.9	78.3
70	67.2	+3.6	−4.7	79.2

**Table 5:** Archival functional checks retained from the submitted manuscript. These are initial checks rather than new controlled reliability trials; controlled HU108 results are reported separately in Section 3.8.

Item	Test Conditions	Recorded Result
Buttons	20 consecutive brief presses	20 responses
Routine ventilation	10 breaths	10 detections
Weak airflow	Reduced breath intensity	One failed trigger; total trials not recorded
Bluetooth	Open line of sight, approx. 10 m; 60 s	Approx. 120 frames; no obvious garbled characters

**Table 6:** Direct electrical step-to-LED-driver benchmark (100 triggers) and mixed-source engineering budget, in milliseconds. Range is maximum minus minimum, not a standard deviation. The input is injected at AD620 and the output is a GPIO transition.

Statistic	Direct Benchmark (ms)	Engineering Budget (ms)
Mean	26.4	26.0
Minimum	19.7	19.3
Maximum	37.2	36.7
Maximum − minimum	17.5	17.4

**Table 7:** Laser-reference positive scenarios. Values are mean ± sample SD across ten 60 s trials on one installation. FN is reference minus TP; irregular FN and sensitivity use the reported mean reference count only. Event-level matching criteria remain to be specified.

Condition	Reference Events/Trial	TP/Trial, Mean ± SD	FN/Trial, Mean	Sensitivity, %	FNR, %
60 cpm	60	54.7 ± 2.16	5.3	91.2	8.8
80 cpm	80	74.8 ± 1.32	5.2	93.5	6.5
120 cpm	120	105.6 ± 2.27	14.4	88.0	12.0
Irregular 60–120 cpm	90 (mean)	79.7 ± 2.21	10.3 *	88.6 *	11.4 *

* For the irregular-rhythm condition, the mean reference count was 90 and the mean TP count was 79.7. The displayed FN count is 90 − 79.7 = 10.3, sensitivity is 100 × 79.7/90 = 88.6%, and FNR is 100 − 88.6 = 11.4%. These are aggregate calculations from reported means, not means of individual-trial sensitivities.

**Table 8:** Invalid-action and resting scenarios. Values are mean ± sample SD across ten 60 s trials on one installation. FPR and specificity are reference-action-normalised count estimates for the two 60-action conditions only; no resting event denominator was supplied.

Condition	Negative Actions/Trial	FP/Trial, Mean ± SD	TN/Trial, Mean	FPR, %	Specificity, %
Shallow (<50 mm)	60	27.9 ± 1.73	32.1	46.5	53.5
Incomplete release	60	12.3 ± 2.21	47.7	20.5	79.5
Rest (60 s)	Not defined	7.4 ± 1.71/min	Not defined	Not defined	Not defined

**Table 9:** Recorded HU108 response to four forward-flow syringe conditions, 30 pulses each. Counts were supplied explicitly in the addendum. No extra false triggers were reported in these positive-pulse blocks; no-flow window specificity is a separate endpoint. Nominal volume and approximate peak flow covaried.

Volume mL	Peak Flow L/min	Pulses	Detected	Missed	Detection %
500	30	30	30	0	100
300	18	30	27	3	90.0
200	12	30	20	10	66.7
100	6	30	3	27	10.0

**Table 10:** Internally consistent laser-reference calibration-set classification results, 120 cycles per condition. Exact 50 and 60 mm values belong to the target class. Higher-depth matrices are withheld from accepted results pending reconciliation in Table S18.

Set Travel (mm)	Cycles	Wrong Classes	Error (%)
40	120	0	0.0
50	120	14	11.7

**Table 11:** Quantitative context from existing CPR feedback studies and the present prototype. Published outcomes retain their original endpoint definitions and are not a same-condition device comparison.

System and Evaluation	Reported Quantitative Result	Evidence Boundary
Smartphone accelerometer [8] 60 sessions of 30 s	Depth agreement reported within ±4 mm; compression-count agreement coefficient 0.92 (95% CI 0.88–0.95).	Compared with a standard CPR quality tool. The agreement endpoint differs from MAE and RMSE.
Smartwatch feedback [9] 30-participant crossover study	With feedback versus unaided compressions, depth effectiveness was 68.4% versus 29.7%; rate effectiveness was 87.5% versus 30.1%.	Rescuer performance within the study-defined target ranges, not displacement error.
Present prototype Five static system/laser pairs	System-minus-laser bias +1.14 mm; MAE 1.34 mm; RMSE 1.98 mm. Descriptive agreement lines −2.40 to +4.68 mm.	Pairs also used for recalibration. No held-out or commercial-device comparison.
Present prototype Controlled airflow and electrical input	Routine/weakest pulse detections 30/30 and 3/30; no-flow window specificity 99.89%. Mean electrical input-to-driver delay 26.4 ms (100 triggers).	Binary airflow events and electrical driver timing; no tidal-volume accuracy or physical compression-to-light/sound latency.

## Data Availability

The accompanying revision package contains English source tables, individual trial counts where retained, Supplementary Information and a result-to-source provenance map. Table 3, Table 4 and Table 5 and the numerical inputs to Figure 6 and Figure 7 are reproduced by code/reproduce_results.py from data/revised_static_pairs.csv, data/original_dynamic_summary.csv and data/original_functional_checks.csv. Figure presentation is supplied in figures/CPR_Editable_Figures.opju, with editable worksheets, exports and Origin rebuilding scripts. The original and revised 8 kg comparisons remain separate, and the author’s batch clarification is recorded in the provenance notes. Complete acquisition waveforms and all cycle-level reference/output records are unavailable. No public repository identifier has been assigned. Supplementary Tables S1–S22, Figures S1–S4 and Notes S21–S22 are supplied in the separate Supplementary Materials file. The package includes executable summary-analysis and plotting code and an archived STM32 Keil project. The firmware README documents the settings and differences from the declared processing model. The archive supports source inspection; its correspondence to every generating build and its operation in a hardware rebuild remain unverified. Missing acquisition records and comparator firmware are identified in the package documentation.

## Supplementary Materials

The following supporting information can be downloaded at: https://www.mdpi.com/article/10.3390/s26216880/s1: Table S1. Loading and unloading at laser-reference displacements; Table S2. Position-specific recalibration; Table S3. Three independent installations after recalibration; Table S4. Algorithm comparison by set travel; Table S5. Algorithm comparison by compression rate; Table S6. Boundary-case summaries and unresolved ventilation definitions; Figure S1. Rate and boundary-case comparisons; Table S7. Individual true-positive counts in laser-reference valid-compression conditions; Table S8. Individual false-positive counts in invalid-action and resting conditions; Figure S2. Qualified-compression performance by condition; Table S9. Original calibration reconstruction and local sensitivity; Table S10. Additional author reported diagnostics at 8 kg; Table S11. Controlled HU108 response; Figure S3. Controlled airflow response and laser depth classification; Table S12. Additional mixed source timing estimates by rate; Table S13. Reported 100 cpm stage budget; Table S14. Uploaded distribution-derived boundary estimates; Table S15. Direct electrical step to GPIO benchmark; Table S16a. Calibration-set depth-class summary; Table S16b. Reported paired means and peak spans; Figure S4. Reported timing estimates and range widths; Table S17. Individual no-flow test blocks; Table S18. Latest supplied classification matrices and consistency checks; Table S18a. Supplied matrix at 40 mm; Table S18b. Supplied matrix at 50 mm; Table S18c. Supplied matrix at 60 mm; Table S18d. Supplied matrix at 70 mm; Table S18e. Supplied matrix at 50 + 60 mm; Table S19. Illustrative rate definitions; Table S20. Result provenance and reproduction scope; Note S21. Firmware and source-document differences; Note S22. Calibration evidence and measurement uncertainty; Table S21. Available calibration evidence and outstanding uncertainty records; Table S22. Author-declared historical parameters and firmware correspondence.
