# Supplementary material for: Dual-Channel Sensing and Event-Based Feedback for Cardiopulmonary Resuscitation Training
Source: Sensors (Basel). 2026 Oct 30;26(21):6880. doi: 10.3390/s26216880 (PMC13766513; doi:10.3390/s26216880)
Supplement: Supplementary file 1 [file sensors-26-06880-s001.zip › sensors-4674170-supplementary.pdf]

Supplementary Materials

Dual-Channel Sensing and Event-Based Feedback for Cardiopulmonary Resuscitation Training

Manuscript sensors-4674170

Reference numbers correspond to the reference list in the main manuscript. Supplementary labels and released source-file names retain their original identifiers.

Supplementary Table S1. Loading and unloading at laser-reference displacements.

Mass<br>kg	Laser<br>mm	Loading<br>mm	Unloading<br>mm	Separation<br>mm	Relative<br>%	*K* load<br>mm/count	*K* unload<br>mm/count	*K* change<br>%
2	19.2	18.8	19.5	0.7	3.65	0.098	0.102	4.08
4	38.5	37.8	38.9	1.1	2.86	0.099	0.102	3.03
6	57.7	58.5	59.9	1.4	2.43	0.101	0.104	2.97
8	76.9	72.6	74.5	1.9	2.47	0.094	0.097	3.19
10	96.2	94.8	97.1	2.3	2.39	0.098	0.101	3.06

Branch separation is unloading minus loading. Relative separation divides by the corresponding laser displacement. Across the five depth points, mean separation was 1.48 mm and mean relative separation approximately 2.76%. Direction-specific K values reproduce the supplied summary. Their means are 0.0980 and 0.1012 mm/count. Cycle counts and the raw ADC/fitting procedure remain to be documented. No independent sample size is inferred from five means.

Supplementary Table S2. Position-specific recalibration.

Position	Refitted *K*<br>mm/count	Zero<br>counts	Static MAE<br>mm	Static RMSE<br>mm	50 mm bias<br>mm	Within ±3 mm<br>%	*K* change<br>%
A_center	0.1	1245.0	1.52	2.09	-1.0	83.3	0.0
B_left_20mm	0.0922	1228.0	2.14	2.76	-2.3	71.7	-7.8
C_up_20mm	0.0956	1237.0	1.87	2.41	-1.6	77.5	-4.4

A is the reference centre, *B* is 20 mm to the left and C is 20 mm upward. Errors are the reported values after each position was refitted. The centre MAE 1.52 mm and RMSE 2.09 mm belong to this summary and are not substituted for the revised five-pair values in the main Table 3. *The t*rial counts and whether evaluation points were used in fitting remain unspecified.

Supplementary Table S3. Three independent installations after recalibration.

Install	Refitted *K*<br>mm/count	Zero<br>counts	Static bias<br>mm	Static RMSE<br>mm	50 mm mean<br>mm	Within ±3 mm<br>%	*K* change<br>%
1	0.1	1245.0	-1.2	2.09	49.0	83.3	0.0
2	0.0982	1262.0	-1.47	2.25	48.5	80.8	-1.8
3	0.1015	1231.0	-0.93	2.17	49.4	82.1	1.5

Across three installation-level entries, $K = 0.0999 \pm 0.00165$ mm/count (mean ± sample SD), relative SD 1.65%; zero = 1246 counts, relative SD 1.25%; RMSE = 2.17 ± 0.08 mm, relative SD 3.69%. The source RMSE relative SD of 3.83% was corrected from the three supplied RMSE values. These are between-installation summaries after refitting, not uncertainty intervals for an individual compression.

Supplementary Table S4. Algorithm comparison by set travel.

Travel mm	Method	Cycle accuracy %	Vent. matching %	FP cycles /120	FN vent. /120	Phase error %	Mean latency ms
40.0	Current	98.3	96.7	2.0	0.0	2.5	33.5
40.0	Comparator	92.5	84.2	9.0	2.0	10.8	152.3
50.0	Current	99.2	97.5	1.0	0.0	1.7	33.5
50.0	Comparator	94.2	87.5	7.0	1.0	8.3	151.7
60.0	Current	99.2	97.5	1.0	0.0	1.7	34.0
60.0	Comparator	95.0	88.3	6.0	1.0	7.5	150.9
70.0	Current	98.3	96.7	2.0	0.0	2.5	35.0
70.0	Comparator	93.3	85.8	8.0	2.0	9.2	151.2

Current and Comparator reproduce the supplied method labels. FP and FN count columns retain the source denominators; these denominators do not establish the definitions of the separate percentage columns. Across the four rows for each algorithm, reported FP cycles were total 6 versus 30 and FN ventilation counts 0 versus 6. Trial-level pairing, independent blocks, comparator implementation, and latency start/end events remain to be specified. Differences are descriptive; no p values are calculated.

Supplementary Table S5. Algorithm comparison by compression rate.

Rate cpm	Method	Cycle accuracy %	Vent. matching %	Rate error* cpm	Phase error %
80.0	Current	97.5	95.8	±0.6	3.3
80.0	Comparator	90.8	81.7	±1.9	12.5
100.0	Current	99.2	97.5	±0.4	1.7
100.0	Comparator	94.2	87.5	±1.5	8.3
120.0	Current	98.3	96.7	±0.5	2.5
120.0	Comparator	91.7	83.3	±2.1	11.7

*The ± entries are retained as reported. The source does not define them as SD, limits, mean absolute errors or instrument uncertainty. The common 100 cpm/50 mm percentage values in Tables S4 and S5 may refer to the same condition; these tables are not pooled as independent replications.

Supplementary Table S6. Boundary-case summaries and unresolved ventilation definitions.

Scenario	Method	Cycle accuracy %	Vent. rate† %	FN vent. /10	FP vent. /10	Timing error %
Weak airflow	Current	99.2	90.0	1.0	0.0	3.3
Weak airflow	Comparator	93.3	70.0	3.0	1.0	13.3
Shallow compression	Current	92.5	95.0	0.0	0.0	5.8
Shallow compression	Comparator	78.3	82.5	0.0	2.0	15.0
Incomplete recoil	Current	95.0	96.7	0.0	0.0	4.2
Incomplete recoil	Comparator	80.8	84.2	0.0	1.0	12.5

†Ventilation rates reproduce the source without reconciliation. For shallow compression and incomplete recoil, values below 100% coexist with zero missed events per ten; thus, these columns cannot all be interpreted using one simple TP/(TP+FN) definition. The ventilation percentages are not used as validated detection rates in the main results. The weak-airflow source describes a 30% reduction in tidal volume, but the measurement method was not provided, so the figure uses the qualitative reduced-airflow label. The original recoil setting of 15 mm is retained only as a source condition; released travel versus residual depth must be clarified. No unknown denominator or confusion-matrix entries were imputed.

Supplementary Figure S1. Rate and boundary-case comparisons.

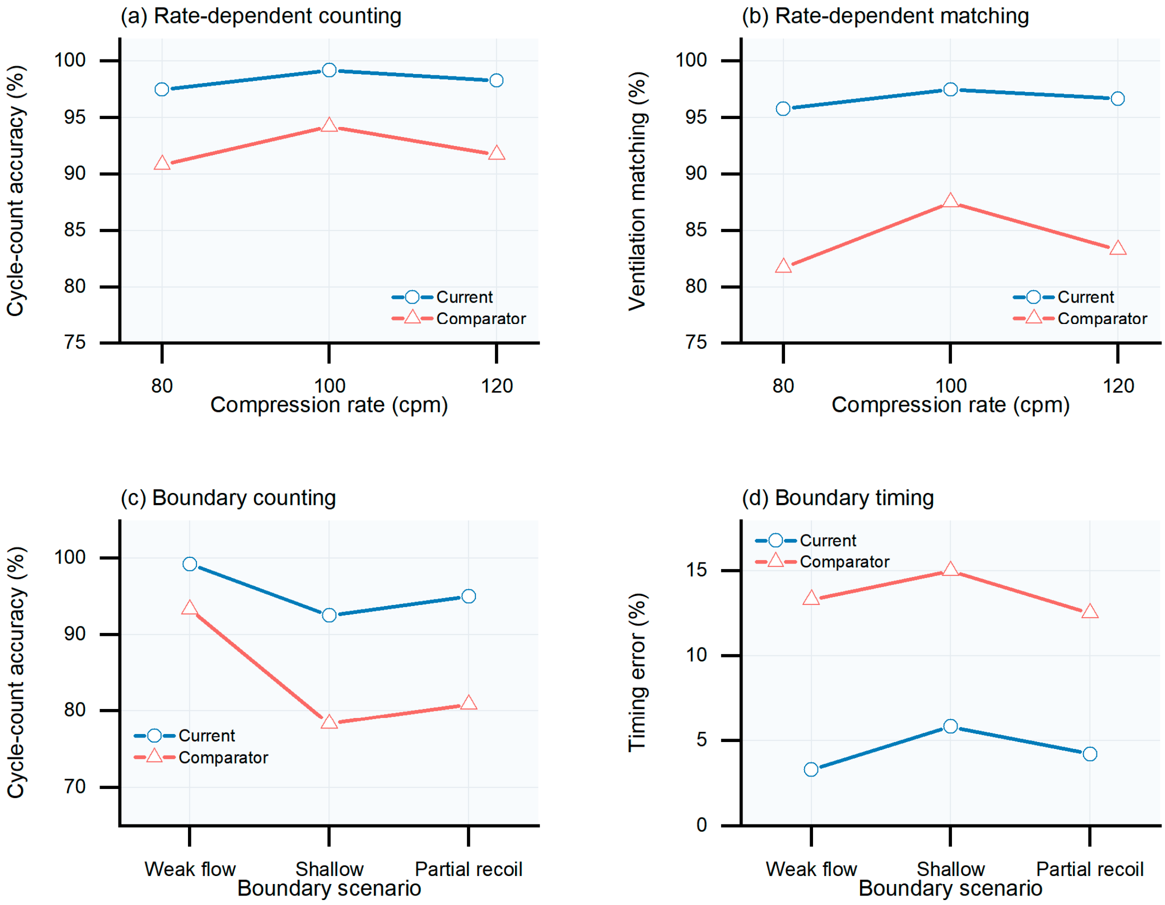

Figure S1. (a,b) Reported cycle-count accuracy and ventilation matching at three compression rates. (c,d) Reported cycle-count accuracy and timing error in weak-flow, shallow-compression and partial-recoil conditions. These are condition summaries, with no repeat-level uncertainty estimates. Lines are visual guides. Tables S5–S6 provide the source values; unreconciled boundary ventilation percentages are excluded.

Definitions and interpretation

Percentage-point differences compare like-labelled endpoints within a source table. The six tables contain summary measurements, not continuous signals or event-level reference labels. They support descriptive comparisons, but do not establish a causal advantage of shared event-state integration, a universal conversion coefficient, or a clinical ventilation-volume assessment. Gauge configurations were recalibrated. Independent validation requires frozen conversion parameters and a documented separation of fitting and test data.

Supplementary Table S7. Individual true-positive counts in laser-reference valid-compression conditions.

trial	TP_60_cpm	TP_80_cpm	TP_120_cpm	TP_irregular_mean90_reference
1	54	75	107	81
2	53	74	104	78
3	54	76	106	80
4	55	75	108	82
5	51	73	103	77
6	57	77	107	81
7	58	76	109	83
8	53	74	104	79
9	57	75	106	80
10	55	73	102	76

Each cell is the reported qualified-compression true-positive count in a 60 s trial. Reference event counts were 60, 80 and 120 in the regular-rhythm columns; only the mean reference count (90) was supplied for irregular rhythm. These are ten repeated trials on one installation. No event timestamps or matching tolerance were supplied.

Supplementary Table S8. Individual false-positive counts in invalid-action and resting conditions.

trial	FP_shallow_per_60	FP_partial_release_per_60	FP_rest_per_60s
1	28	10	11
2	26	11	8
3	27	12	9
4	31	15	7
5	28	13	8
6	30	14	7
7	25	9	6
8	28	11	5
9	28	12	6
10	28	16	7

The shallow and incomplete-release conditions each had 60 invalid reference actions per 60 s trial. Rest had no specified negative event opportunities; its counts are false outputs per minute. The source standard deviations for 80 cpm and incomplete release have been recalculated from individual observations.

Supplementary Figure S2. Qualified-compression performance by condition.

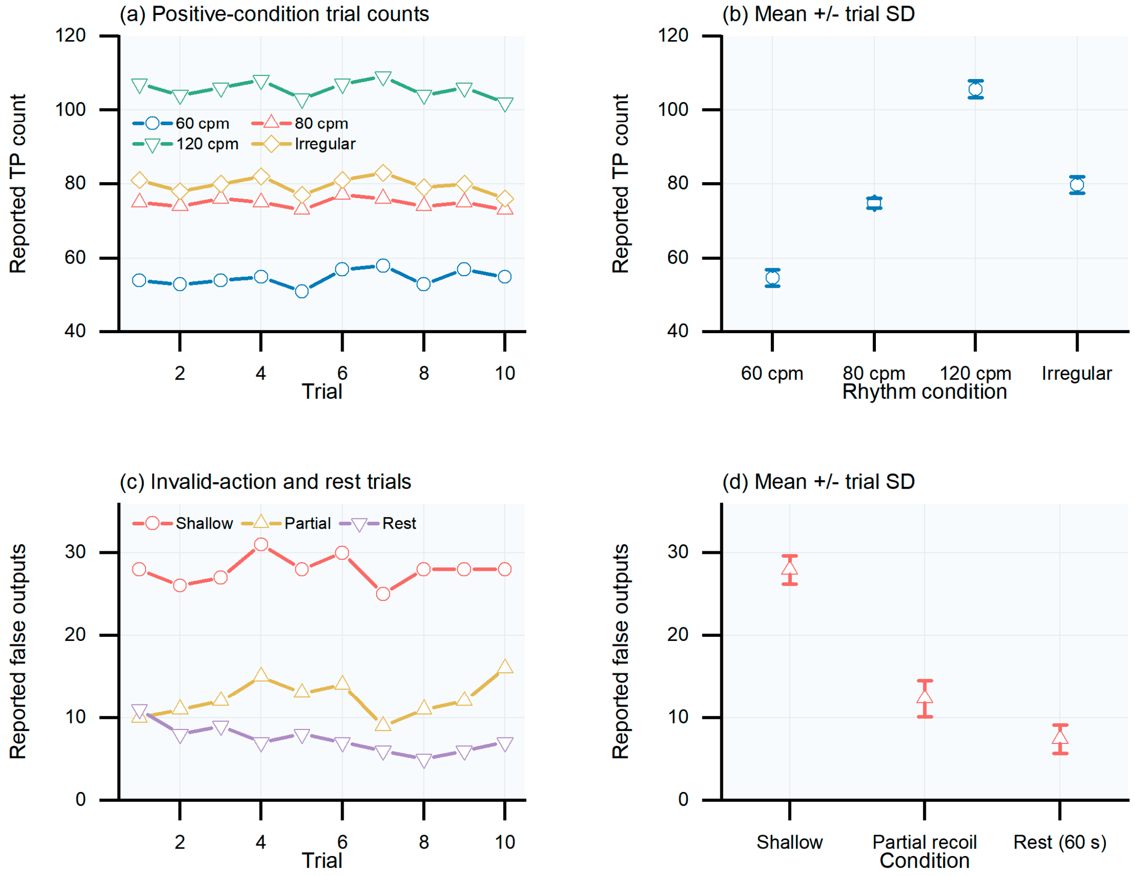

Figure S2. Retained counts from ten technical repeats on one installation. (a) Reported TP counts for 60, 80 and 120 cpm and irregular rhythm. (b) Means with sample-SD error bars for these counts. (c) False qualified outputs in shallow, partial-recoil and resting trials. (d) Corresponding means with sample-SD error bars. Shallow and partial-recoil trials each contain 60 reference actions; resting outputs are per 60 s. The irregular reference count is available only as a mean of 90, so individual TP counts are not divided by a reconstructed reference sequence. Tables S7–S8 contain all trial values; no event-matched or between-installation accuracy is implied.

Supplementary Table S9. Original calibration reconstruction and local sensitivity

The author confirmed that the five A values below were independently recorded zero-referenced ADC magnitudes, rather than values reconstructed from the depth column. $A = |\bar{X} - X_0|$. The original setting $K = 0.1000$ mm/count and $\Delta_0 = 90$ counts reconstructs the original Table 3 estimates. These are separate from the revised Table 3 laser pairs. No per-trial ADC records or zero-level values were provided.

Mass kg	Reference mm	A counts	A−90 counts	Estimated mm	Reference-equivalent A counts	Residual mm
2	19.2	278	188	18.8	282	-0.4
4	38.5	468	378	37.8	475	-0.7
6	57.7	675	585	58.5	667	+0.8
8	76.9	816	726	72.6	859	-4.3
10	96.2	1038	948	94.8	1052	-1.4

Reference-equivalent A is $d_{ref}/K+\Delta_0$, a calculated model input rather than a separate measurement. At 8 kg, A−Aref = 816–859 = −43 counts and $K(A-\text{Aref}) = -4.3$ mm. The corresponding output discrepancy is −5.59% of 76.9 mm. This equality is an algebraic explanation of the residual and does not by itself identify its physical cause.

The reference-depth secant slopes are 0.13617 mm/count for 6–8 kg, 0.08694 mm/count for 8–10 kg and 0.10606 mm/count for 6–10 kg. Using the estimated-depth column instead yields $K = 0.1000$ for every interval by construction. The near-linear reference-depth versus mass relation is $d_{ref} = 9.620m-0.020$ mm, $R^2 = 0.9999989$, based on the five rounded reference values. This descriptive fit does not establish metrological traceability or validate the local strain-to-depth conversion.

With zero and dead zone fixed, the through-origin least-squares fit to $z=A-90$ and d_{ref} gives $K_{LS} = \Sigma z\text{dref}/\Sigma z^2 = 0.10222008$ mm/count. Its 8 kg output is 74.2118 mm and residual −2.6882 mm; five-point RMSE is 1.5587 mm. This is an exploratory refit of the original calibration values, not an independently validated correction or the new Table 3 calibration. The recorded $K = 0.1000$ reproduces the original outputs but is not the exact minimiser for these pairs. Segment boundaries and the objective are needed to verify the separate reported 1.2 mm global/segmented difference.

At fixed A and K in the active branch, a dead-zone change $\delta\Delta_0$ changes depth by $-K\delta\Delta_0$. Thus 90 counts correspond to a 9.0 mm subtraction at $K = 0.1000$. The dead zone cannot simply be ignored at 8 kg, and its interaction with fitted zero/scale must be specified. The 1.9 mm branch difference in Table S1 is an observed directional separation. Relative to 76.9 mm, the loading residual is −4.3 mm and unloading residual −2.4 mm, giving −4.3 = −2.4−1.9 mm. This identity is not a decomposition into independently measured physical mechanisms.

Supplementary Table S10. Additional author reported diagnostics at 8 kg

The following approximate magnitudes and methods were supplied by the author. They are retained as separate diagnostics. Raw paired readings, per-cycle observations, fit definitions and signed position-level differences were not supplied, so no uncertainty estimates or summed causal allocation are inferred.

Diagnostic	Magnitude and direction	Author supplied method	Interpretation limit
Local strain/displacement comparison	~1.8 mm; negative	Laser displacement versus strain-equivalent depth at the same load; 3 repeated loadings per point; 60 s hold before sampling.	The comparison remains a total residual unless other effects are separately controlled. Paired readings and separation procedure are not supplied.
Global versus segmented calibration	~1.2 mm; reported negative	Mathematical comparison using five fixed ADC/reference-depth pairs. No additional physical repetitions.	Segment boundaries, fit objective and held-out points are unspecified. The reported value is not reproduced by the fixed-dead-zone least-squares diagnostic in Table S9.
Loading/unloading diagnostic	~0.7 mm; reported negative on loading	3 complete loading/unloading cycles at each point, according to the added author description.	This magnitude differs from the 1.9 mm branch separation at 8 kg in Table S1. Its relation to that dataset requires clarification; branch separation is not a uniquely isolated friction or creep contribution.
Load-position diagnostic	~0.4 mm; reported negative	8 kg at centre, 10 mm left and 10 mm forward; 5 repeated placements at each location.	The original CSV instead describes approximately 5 mm eccentricity. Confirm which offset produced 0.4 mm and provide the position-level readings; keep separate from the 20 mm gauge-placement tests in Table S2.
Loaded signal fluctuation	~0.2 mm; bidirectional, reported zero mean	3 continuous 60 s ADC recordings per point at fixed load and location; reported statistic is peak-to-peak equivalent-depth fluctuation.	Peak-to-peak fluctuation is neither a signed bias nor a standard uncertainty and cannot be added as a negative contribution. Raw time series and averaging conditions are needed.

The five reported magnitudes sum arithmetically to 4.3 mm, but the final entry is a zero-mean, bidirectional peak-to-peak fluctuation and the other entries are not demonstrated to be mutually exclusive effects. Neither arithmetic summation nor root-sum-of-squares propagation constitutes a validated bias budget for these heterogeneous quantities. Reconciliation of the loading/unloading and load-offset definitions is required before causal attribution.

Supplementary Table S11. Controlled HU108 response

Volume mL	Approx. peak L/min	Inputs	Detected	Missed	Extra triggers	Detection %
500	30	30	30	0	0	100.0
300	18	30	27	3	0	90.0
200	12	30	20	10	0	66.7
100	6	30	3	27	0	10.0

The addendum supplies these detected/missed counts explicitly. All inputs were forward-inspiratory syringe pulses. The zero extra-trigger column records positive-pulse blocks and does not supply a negative-event denominator. Window specificity is calculated from the separate no-flow blocks in Table S17. Volume and peak flow were varied together.

Ten rising-flow threshold tests gave a reported mean switch-on flow of 12.5 L/min and CV 4.2%. Three 5 min no-flow blocks yielded one false falling edge during 15 min, or 0.0667 false events/min. Using the author-defined non-overlapping 1 s windows, specificity is 899/900 = 99.8889%, reported as 99.89% (99.9% at one decimal), rather than the source 99.8%. Window specificity differs from event-pulse sensitivity and from the proportion of time spent at a low output level. Sequential windows are not independent installations.

Reference setup and tested direction

The gradient-volume tests used a calibrated 1 L syringe with stated volume accuracy ±0.5%, with all pulses applied in the forward inspiratory direction. A reference flowmeter with stated range 0–120 L/min and accuracy ±2% was upstream of HU108 in one unbranched gas path. Reference-flow and switch-level signals were recorded with stated 1 ms synchronisation. The four settings combined 500, 300, 200 and 100 mL with approximate peak flows of 30, 18, 12 and 6 L/min, respectively, with 30 pulses per setting. Detected, missed and additional-trigger counts were recorded explicitly. Ten rising-flow tests characterised the switch-on threshold. Three 5 min no-flow blocks provided 900 non-overlapping 1 s windows; a window containing a false falling edge was positive. Specificity was TN/(TN+FP). These sequential windows are not independent installations. Reverse-flow, leakage and waveform-distortion tests were not performed.

Supplementary Figure S3. Controlled airflow response and laser depth classification

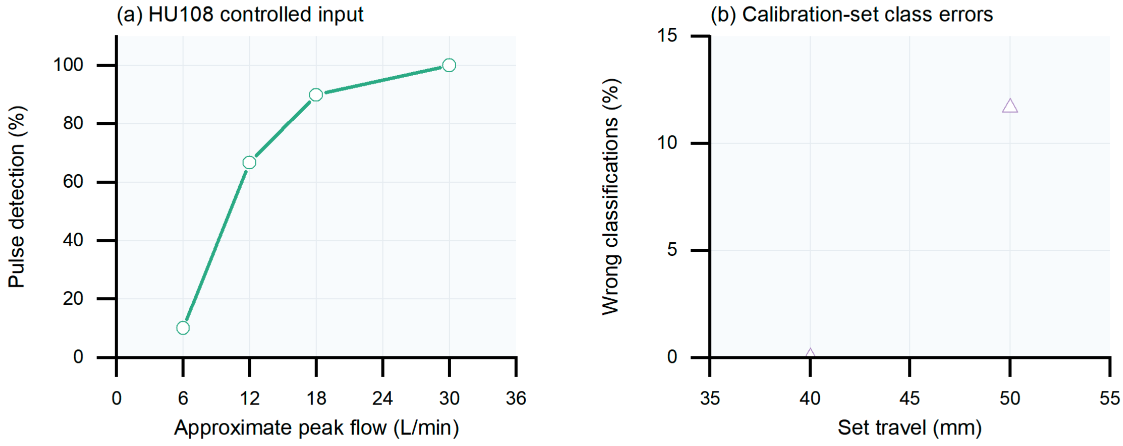

Figure S3. (a) Controlled HU108 pulse detection at four approximate peak-flow conditions, with 30 forward-inspiratory pulses each. Peak flow and nominal volume covaried. The points are observed fractions and the line is a visual guide, not a fitted threshold curve. (b) Accepted calibration-set classification errors at 40 and 50 mm, each with 120 cycles. *K* and the dead zone were fitted on the evaluated data. No switch-threshold reference line, inferred error bars or unresolved 60/70 mm error rates are plotted. Tables S11, S16 and S18 document the source and excluded conflicting entries.

Supplementary Table S12. Additional mixed source timing estimates by rate

Rate<br>cpm	Peak mean<br>ms	Peak width<br>ms	Count mean<br>ms	Original count width<br>ms	Reconciled count width<br>ms
60	25.2	17.1	32.2	17.1	Pending
80	25.6	17.2	32.6	17.2	Pending
100	26	17.4	33	17.4	22.4
120	26.5	17.7	33.5	17.7	Pending
140	27.1	18	34.1	18	Pending

The addendum explicitly identifies this series as engineering-derived, not synchronised per-event end-to-end observations. The 120 cycles per rate describe workload context. Original count-range entries are preserved for provenance, but their reconciliation after the 5–10 ms release correction is available only at 100 cpm. They are not SD, confidence intervals, or observed jitter distributions.

Supplementary Table S13. Reported 100 cpm stage budget

Stage	Mean<br>ms	Min–max<br>ms	Width<br>ms
Analogue front end	5	4–6	2
ADC schedule offset	2.5	0–5	5
Four-sample processing allowance	10	10–10	0
Peak-state detection	8	5–15	10
Local driver IO	0.5	0.3–0.7	0.4
Peak-feedback budget	26	19.3–36.7	17.4
Release-confirmation allowance	7	5–10	5
Count-confirmation budget	33	24.3–46.7	22.4
Bluetooth send schedule	250	0–500	500
Bluetooth transport allowance	20	18–22	4
Bluetooth total budget	303	42.3–568.7	526.4

This is a mixed-source engineering budget. The release allowance is now nominal 5 ms debounce plus an estimated 0–5 ms crossing interval, giving minimum/mean/maximum 5/7/10 ms. At 100 cpm the count budget is 33 ms mean and 24.3–46.7 ms range (22.4 ms width). The Bluetooth budget is 303 ms mean and 42.3–568.7 ms range (526.4 ms width). These are arithmetic consequences of the corrected allowance, not new measurements. The old maxima 41.7 and 563.7 ms are superseded for this budget. A four-tap average at 5 ms spacing still has a 7.5 ms ideal group delay and 15 ms first-to-fourth-sample span; 10 ms is only an engineering processing allocation. Stage time anchors and their independence still require records before a strict serial decomposition can be verified.

Supplementary Table S14. Uploaded distribution-derived boundary estimates

Set travel<br>mm	Nominal<br>cycles	Estimated errors<br>50 mm	Estimated rate<br>%	Estimated errors<br>60 mm	Estimated rate<br>%
40	120	2	1.7	0	0.0
50	120	14	11.7	3	2.5
60	120	4	3.3	18	15.0
70	120	0	0.0	5	4.2

These are the original uploaded CSV values, clarified by the author as distribution-derived estimates. They are not empirical paired error counts. The Gaussian variance, fitting rule and individual depth records were not supplied, and a mean and observed range do not uniquely determine a Gaussian model. These entries do not override Table S16 or the observed extrema in original the main Table 4. No claim of less than 1.5% agreement with the laser results is made because the compared endpoints and datasets differ.

Supplementary Table S15. Direct electrical step to GPIO benchmark

Statistic	Observed<br>ms	Budget<br>ms	Relative difference<br>%
Mean	26.4	26	+1.54
Minimum	19.7	19.3	+2.07
Maximum	37.2	36.7	+1.36
Peak-to-peak	17.5	17.4	+0.57

The author supplied this additional benchmark in the revision discussion. There were 100 electrical steps. The reference was the 50% rising-edge point of a differential step injected at the AD620 input; the output was the 50% transition of the LED-driving GPIO. The source was specified with a <1 μs rise time and <0.1% amplitude error. The oscilloscope was specified as 100 MHz bandwidth and 1 ns time resolution, the logic analyser as 1 GHz sampling. Instrument resolution is not the measurement uncertainty. The test bypasses the manikin and strain bridge and does not measure photodiode or microphone onset. Mean agreement with the mixed-source budget is 1.54%; the minimum differs by 2.07%, so the blanket “all below 2%” claim is not retained. The measured maximum is 0.5 ms above the budget maximum.

The latest addendum confirms that cycle-count and Bluetooth totals have not received direct full-chain end-to-end measurement. Earlier statements about less than 3% or 5% agreement are not used to validate these totals without paired records. The 5–10 ms release increment is a nominal engineering allowance. Approximately 120 Bluetooth frames in 60 s describe cadence rather than one-way message latency.

Supplementary Table S16a. Calibration-set depth-class summary

Set travel<br>mm	Mean laser<br>mm	Cycles	Accepted wrong classes	Accepted error<br>%
40	40.1	120	0	0.0
50	49.8	120	14	11.7
60	58.1	120	Pending	Pending
70	67.4	120	Pending	Pending

The author clarified that K and the dead zone were fitted using these evaluation data. The results are calibration-set agreement, not held-out accuracy. The latest 40 mm matrix is diagonal (0 errors), and the 50 mm matrix has 14 errors. These supersede earlier 2/16/21/4 and 2/14/18/4 error-count versions. The 60 and 70 mm error rates are withheld because the latest cells, stated totals and reported ranges are inconsistent; all supplied matrices are retained in Table S18.

Supplementary Table S16b. Reported paired means and peak spans

Set<br>mm	Laser mean<br>mm	System mean<br>mm	Mean bias<br>mm	Laser span<br>mm	System span<br>mm
40.0	40.1	39.2	-0.9	6.6	6.5
50.0	49.8	49.0	-0.8	7.9	7.8
60.0	58.1	58.3	0.2	9.7	9.5
70.0	67.4	67.2	-0.2	8.3	8.1

Mean biases are the differences between the supplied means, not MAE/RMSE from paired cycles. The reported spans are retained as source summaries, not validated full-sample bounds. In the latest 60 mm matrix the predicted classes include both <50 and >60 mm, which requires a system span >10 mm, inconsistent with 9.5 mm. At 70 mm, reference target-class entries conflict with the stated minimum of 63.3 mm. The displayed rounded extrema 63.3–71.5 differ by 8.2 mm versus a reported span of 8.3 mm; independent rounding could explain that 0.1 mm difference, so original precision should be checked. Paired records are needed to align the summaries.

Supplementary Figure S4. Reported timing estimates and range widths

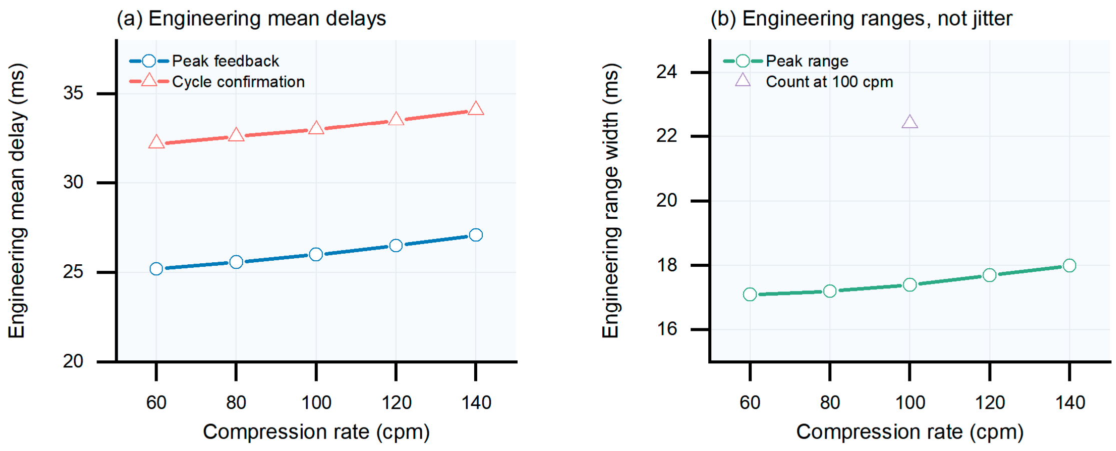

Figure S4. (a) Engineering-derived mean delay allocations across five workload rates. (b) Engineering peak-delay range widths, with the single reconciled count-delay width of 22.4 ms at 100 cpm. The other count-range entries remain unreconciled and are not plotted. Lines are visual guides. These ranges are not measured jitter distributions, and the stated 120 cycles per rate are workload context rather than supplied synchronous timing pairs. The direct 100-step electrical benchmark is separate in Table S15.

Supplementary Table S17. Individual no-flow test blocks

Block	Duration min	False edges	Event time within block	Approx. low duration ms
1	5	0	None	—
2	5	1	3 min 12 s	120
3	5	0	None	—
Total	15	1		

There are 900 non-overlapping one-second negative windows, with 899 true-negative and one false-positive window. Specificity is 99.8889%, reported as 99.89% or 99.9% to one decimal. The original addendum's "99.89% rounded to 99.8%" wording is a rounding error. The low-level duration is not substituted for the event-window denominator. Reverse-flow, leakage and distorted-waveform tests remain absent.

Supplementary Table S18. Latest supplied classification matrices and consistency checks

Table S18a. Supplied matrix at 40 mm

Prediction / laser reference	Shallow <50	Target 50–60	Deep >60	Row total
Predicted shallow	120	0	0	120
Predicted target	0	0	0	0
Predicted deep	0	0	0	0
Column total	120	0	0	120

Total 120; diagonal 120; off-diagonal 0. This is consistent with the reported below 50 mm depth ranges and supersedes the earlier two-event classification entry.

Table S18b. Supplied matrix at 50 mm

Prediction / laser reference	Shallow <50	Target 50–60	Deep >60	Row total
Predicted shallow	52	3	0	55
Predicted target	11	54	0	65
Predicted deep	0	0	0	0
Column total	63	57	0	120

Total 120; diagonal 106; off-diagonal 14 (11.67%). With ≥50 mm as positive, TP54, FP11, FN3 and TN52. Sensitivity is 54/57 = 94.74% and specificity 52/63 = 82.54%, describing this calibration set.

Table S18c. Supplied matrix at 60 mm

Prediction / laser reference	Shallow <50	Target 50–60	Deep >60	Row total
Predicted shallow	0	5	0	5
Predicted target	0	88	10	98
Predicted deep	0	4	13	17
Column total	0	97	23	120

Total 120; diagonal 101; off-diagonal 19 (15.83% arithmetically), not the stated 14. The upper-boundary-only error is 10+4=14, while five predicted-shallow entries add further three-class errors. Predicted shallow and deep categories require a system peak span >10 mm, contradicting the reported 9.5 mm span. No verified error rate is assigned.

Table S18d. Supplied matrix at 70 mm

Prediction / laser reference	Shallow <50	Target 50–60	Deep >60	Row total
Predicted shallow	0	0	0	0
Predicted target	0	5	0	5
Predicted deep	0	0	115	115
Column total	0	5	115	120

Total 120; all 120 are diagonal, so the supplied cells imply zero errors, not the stated five. The reference target-class column contains five entries, contradicting the stated laser minimum of 63.3 mm. No verified error rate is assigned.

Table S18e. Supplied matrix at 50+60 mm

Prediction / laser reference	Shallow <50	Target 50–60	Deep >60	Row total
Predicted shallow	52	8	0	60
Predicted target	11	142	10	163
Predicted deep	0	4	13	17
Column total	63	154	23	240

Total 240; diagonal 207; off-diagonal 33, giving an arithmetic 13.75%. This matches the addition of the two supplied matrices but inherits the unresolved 60 mm conflict. It is not reported as a verified combined error rate.

These matrices reproduce the latest author-supplied entries. The table distinguishes cell arithmetic from validation of the experimental labels. No cell has been moved or altered to force agreement with a narrative total or a depth span. The earlier 140-cycle array and intermediate 240-cycle version are archived only in the source-data history.

Supplementary Table S19. Illustrative rate definitions

Metric	Definition	Illustrative value cpm
Segment-average confirmed-cycle rate	$60(N-1)/(t_N-t_1)$, including intervening pauses	Approximately 92
Active-bout cadence	$60\Sigma(\mathrm{N}j-1)/\Sigma(t_{\mathrm{last},j}-t_{\mathrm{first},j})$, excluding inter-bout intervals	Approximately 100

The values are illustrative examples supplied by the author, not a measured timestamp reanalysis. An actual comparison needs bout boundaries and paired cycle/ventilation times. The 100–120 cpm target refers to active compression cadence; a segment-average value including pauses has a different interpretation.

Supplementary Table S20. Result provenance and reproduction scope

Item	Source and status	Released input	Reproduction scope
Table 3 and Figure 6	New static laser pairs; same data used for recalibration	revised_static_pairs.csv	Five pairs; bias, MAE, RMSE, regression and descriptive Bland–Altman plot
Table 4 and Figure 7	Archival dynamic summaries matching thesis Table 5.2	original_dynamic_summary.csv	Four summaries of 120 cycles; individual cycles unavailable
Table 5	Archival initial functional checks	original_functional_checks.csv	Four categories; weak-flow denominator absent
Tables 7–8	Additional qualified-count trial records	S7_positive_trial_counts.csv; S8_negative_trial_counts.csv	Ten trials per scenario; matching tolerance and generating counter absent
Table 9	Additional controlled forward-flow and no-flow summaries	S11_HU108_event_response.csv; S17_no_flow_blocks.csv	30 pulses per condition and three no-flow blocks
Table 6	Additional direct electrical benchmark	Table6_direct_electrical_benchmark.csv	100-trigger summary; individual time pairs unavailable
Table 10	Consistent subset of additional classification summaries	S18_matrix_40_as_supplied.csv; S18_matrix_50_as_supplied.csv	Calibration-set counts; higher-depth matrices unresolved
Timing by rate	Engineering-derived estimates	S12_additional_latency_by_rate.csv; S13_reported_latency_budget.csv	Budget arithmetic only, not measured end-to-end jitter
Firmware	Archived source project, not version-linked to all experiments	firmware/archived_project/USER/AIWOMA.uvprojx	Source inspection; build/hardware replay not verified
Historical 8 kg static entries	Separate runs on one platform, per author clarification; thesis ADC/zero unavailable.	Original static summary; Table S9; author batch note; thesis Table 5.1.	Both 74.6 and 72.6 mm retained; 816 counts verifies 72.6 mm. No quantified causal allocation.

Supplementary Note S21. Firmware and source-document differences

The original static summaries are preserved as distinct records. Table 5.1 in Liu's thesis [18] reports 74.6 mm at 8 kg, whereas the pre-submission nominal-depth comparison reports 72.6 mm, both against 76.9 mm. According to the retained author batch clarification, these were separate static-loading runs on the same platform, with independently acquired zero levels and the same stated $K = 0.1000$ mm/count and $\Delta_0 = 90$ counts. The recorded 816-count magnitude verifies 72.6 mm through Equation (2). The thesis ADC and zero records are unavailable, so the 2.0 mm difference cannot be apportioned to baseline, placement or temperature. The thesis's stated stiffness of 10.2 N/mm would give 1.92 mm at 2 kg rather than 19.2 mm; this source inconsistency is retained here for traceability.

The analysis programme reproduces the numerical results in Tables 3–5 and the source values for Figures 6–7. The supplied Origin project reproduces the revised figure presentation from the same values. The archived Keil project uses four within-call ADC conversions, an integer depth mapping with a 90-count dead zone, 40–90 mm in-cycle acceptance and an 8 mm release threshold. It has not been version-linked to all experiments and does not supply the separate

qualified-compression counter or matched comparator. Complete continuous acquisition records and all per-event reference/output pairs were not retained. Reproducibility is therefore established for the released summary analysis, without a verified hardware rebuild or replay of every experiment.

Supplementary Note S22. Calibration evidence and measurement uncertainty

The quantities below describe different sources of variation and evidence gaps. They are not interchangeable standard uncertainties and are not summed as an error budget. The original coefficient and resting fluctuation do not establish system depth resolution, particularly when the archived firmware applies integer mapping.

Supplementary Table S21. Available calibration evidence and outstanding uncertainty records

Source	Available evidence	Interpretation	Further record required
Baseline and zero	Zero level 1231–1262 counts across three refitted installations.	Between-installation baseline differences, not longitudinal drift.	Time-stamped unloaded ADC, temperature, supply and zero-acquisition records.
Scale and mounting	K 0.0982–0.1015 mm/count across three installations, sample RSD 1.65%; position shifts −7.8% and −4.4%.	Installation-specific calibration dependence.	Separate calibration and evaluation records for each installation; one fixed K only if transfer is claimed.
Loading direction	Unloading-minus-loading separation 0.7–2.3 mm across five depths.	Directional branch dependence from supplied means.	Repeat-level loading/unloading pairs, cycle order, dwell time and sample count.
Laser-reference agreement	Five recalibration pairs; bias +1.14 mm, MAE 1.34 mm, RMSE 1.98 mm.	Agreement on reused calibration data.	New paired test data after freezing K, zero and dead zone, plus reference calibration and alignment records.
Noise and quantisation	Resting fluctuation 65–80 counts peak to peak; archived integer depth mapping.	Neither RMS uncertainty nor effective depth resolution is established.	Raw ADC series, acquisition bandwidth, voltage reference and generating firmware version.
Time and environment	Earlier drift tables contain expected/component values with unreconciled units.	No validated operating-time or thermal drift coefficient.	Warm-up and temperature-controlled tests at unloaded and fixed-displacement inputs.
Reference and geometry	A separate laser instrument was used; full static-reference documentation is absent.	A physical-reference channel is available, without a complete traceability/uncertainty budget.	Instrument calibration uncertainty, displacement geometry, alignment and batch identifiers.

No combined or expanded uncertainty with a stated coverage probability is assigned. Obtaining such an estimate requires reference-instrument calibration, repeated validation measurements, time/temperature records and the covariance of fitted baseline, dead-zone and scale parameters. Bias, branch separation, between-installation SD and peak-to-peak noise are retained under their own definitions.

Supplementary Table S22. Author-declared historical parameters and firmware correspondence

Parameter	Sym.	Value and unit	Definition and evidence	Source and scope
Unloaded baseline	X_0	1245 ADC counts	Author reports the mean of 32 unloaded readings at 24 ± 1 °C.	Original static-session declaration; individual readings and cited original_static_pairs.csv are not in the inspected release.
Dead zone	Δ_0	90 ADC counts	Fixed engineering threshold for near-zero fluctuations.	Author declaration and archived PRESSURE_RAW_DEADZONE; not a measured uncertainty.
Depth scale	K	0.1000 mm/count	Adopted nominal setting. Fixed-zero/dead-zone least squares on Table S9 gives 0.10222 mm/count.	Historical conversion and author declaration; revised laser-fit constants not established.
Nominal sampling interval	T_s	Approximately 5 ms	Author reports timer-triggered acquisition. The archive instead has a 5 ms main-loop delay.	Timer configuration and version-matched timing records are not supplied. A loop delay is not proof of uniform ADC spacing.
Averaging window	N	4 samples	Declared four-point moving average (a third-order FIR under uniform temporal sampling).	Archive averages four successive ADC conversions within one function call; it does not establish a four-update temporal moving average.
Mechanical-cycle window	—	40–90 mm	Declared candidate-peak window; distinct from the 50–60 mm quality class.	Archive accepts an in-cycle sample in this interval, rather than verifying a peak-only gate.
Release threshold	—	Approximately 8 mm	Low-depth criterion used to confirm release.	Author declaration and archived PRESSURE_RELEASE_THRESHOLD; does not establish complete physical recoil.

The baseline and 24 ± 1 °C condition are author-reported for the historical static session. They neither supply the missing thesis zero/ADC record nor demonstrate thermal stability. The nominal settings are not substituted for the unavailable revised calibration constants. A timer-triggered, temporally averaged implementation requires its own version-linked source and acquisition log.
